# CircHIPK3 regulates pulmonary fibrosis by facilitating glycolysis in miR-30a-3p/FOXK2-dependent manner

**DOI:** 10.7150/ijbs.57915

**Published:** 2021-06-04

**Authors:** Qi Xu, Demin Cheng, Guanru Li, Yi Liu, Ping Li, Wenqing Sun, Dongyu Ma, Chunhui Ni

**Affiliations:** Center for Global Health, Key Laboratory of Modern Toxicology of Ministry of Education, Department of Occupational Medical and Environmental Health, School of Public Health, Nanjing Medical University, Nanjing 211166, China.

**Keywords:** silicosis, glycolysis, circHIPK3, ceRNA, FOXK2

## Abstract

Pulmonary fibrosis develops when myofibroblasts and extracellular matrix excessively accumulate in the injured lung, but what drives fibrosis is not fully understood. Glycolysis has been linked to cell growth and proliferation, and several studies have shown enhanced glycolysis promotes pulmonary fibrosis. However, detailed studies describing this switch remain limited. Here, we identified that TGF-β1 effectively increased the expression of circHIPK3 in lung fibroblasts, and circHIPK3 inhibition attenuated the activation, proliferation, and glycolysis of fibroblasts *in vitro*. Dual-luciferase reporter gene assays, RNA immunoprecipitation (RIP), and RNA pull-down assays showed that circHIPK3 could function as a sponge of miR-30a-3p and inhibit its expression. Furthermore, FOXK2, a driver transcription factor of glycolysis, was identified to be a direct target of miR-30a-3p. Mechanistically, circHIPK3 could enhance the expression of FOXK2 via sponging miR-30a-3p, thereby facilitating fibroblast glycolysis and activation. Besides, miR-30a-3p overexpression or FOXK2 knockdown blocked fibroblast activation induced by TGF-β1 and abrogated the profibrotic effects of circHIPK3. Moreover, circHIPK3 and miR-30a-3p were also dysregulated in fibrotic murine lung tissues induced by silica. Adeno-associated virus (AAV)-mediated circHIPK3 silence or miR-30a-3p overexpression alleviated silica-induced pulmonary fibrosis *in vivo*. In conclusion, our results identified circHIPK3/miR-30a-3p/FOXK2 regulatory pathway as an important glycolysis cascade in pulmonary fibrosis.

## Introduction

Pulmonary fibrosis is the generic term for a broad category of lung diseases that involves inappropriate scar tissue formation in the lungs 1. Many agents can contribute to the initiation and progression of lung fibrosis, including microbial agents, radiotherapeutic and chemotherapeutic agents, and occupational and environmental toxins 2. Silica exposure can cause a complex occupational pulmonary fibrosis disease in workers termed silicosis 3. In China, silicosis remains a major medical burden, however, existing drugs only slow disease progression. Like other fibrosis diseases, silicosis is also characterized by alveolar epithelial cell injury, fibroblast differentiation, and aberrant extracellular matrix (ECM) deposition 4. Correcting these alterations is considered a promising strategy for treating pulmonary fibrosis.

Fibroblast is the primary cell type responsible for promoting fibrosis. In the pathogenesis of pulmonary fibrosis, the myofibroblastic phenotype of activated lung-resident fibroblasts is characterized by increased proliferation and matrix production 5-7. To meet the fast growth and proliferation demand, it is reasonable to assume that myofibroblasts can exhibit aerobic glycolysis as an additional bioenergetic and biosynthetic supply, similar to cancer cells 8-10. Moreover, transforming growth factor (TGF)-β1, the major growth factor involved in fibroblast activation, has been proved to be a trigger of glycolytic reprogramming 11-13. Besides, the interaction between glycolysis and ECM production has also been demonstrated at ECM synthesis and secretion 14, 15. Although it is acknowledged that glycolysis occurs in pulmonary fibrosis, the mechanism driving glycolysis remains largely unknown. FOXK2, a member of the FOX family, has recently been identified as a novel regulator that can promote aerobic glycolysis by upregulating the enzymatic machinery required for this 16. However, the function of FOXK2 has not, to our knowledge, been reported in pulmonary fibrosis.

Circular RNAs (circRNAs) are a special subclass of endogenous non-coding RNAs with covalent and closed structures, thereby, they are more stable than linear RNAs. Most circRNAs are highly conserved across species and exhibit dynamic expression patterns in various physiological and pathological conditions 17, 18. In the past several decades, multiple functions of circRNAs have been identified, and the competitive endogenous RNA (ceRNA) hypothesis is the well-accepted mechanism for the regulatory function of circRNAs. Most circRNAs exist in the cytoplasm and contain many miRNA response elements (MREs) that allow them to competitively bind to miRNAs, thus reducing the functional miRNAs 19. Different from circRNAs, miRNAs are another cluster of small non-coding RNAs that negatively regulate gene expression by mRNA degradation or inhibition of mRNA translation 20. Increasing researchers have focused on the interactions between circRNAs and miRNAs in pulmonary fibrosis. For instance, circTADA2A has been confirmed to function as sponges of miR-526b and miR-203, thus releasing the expression of Caveolin-1 and Caveolin-2 to inhibit lung fibroblast activation and proliferation 21. Another circRNA, CDR1as, could promote epithelial-mesenchymal transition (EMT) during pulmonary fibrosis by binding to miR-7 as a ceRNA 22. Despite these observations, the mechanisms of circRNAs in pulmonary fibrosis initiation and progression are not fully revealed.

In this study, we identified that circHIPK3 was upregulated in fibroblasts stimulated with TGF-β1 and silica-induced murine fibrotic lung tissues. Silencing circHIPK3 could significantly inhibit fibroblast activation *in vitro* and alleviate pulmonary fibrosis progression *in vivo*. The databases online predicted the complementary sequences between miR-30a-3p and circHIPK3/FOXK2, indicating the potential ceRNA network of circHIPK3/miR-30a-3p/FOXK2. Further *in vitro* and *in vivo* data confirmed that circHIPK3 could dramatically enhance FOXK2 expression by sponging miR-30a-3p, thereby contributing to pulmonary fibrosis progression via regulating fibroblast glycolysis and activation. Overall, our results imply that pharmacological approaches aiming at circHIPK3 and its downstream molecules may represent new effective therapeutic strategies in pulmonary fibrosis.

## Materials and Methods

### Animal studies

All animal experiments were approved by the Nanjing Medical University Ethics Committee (Nanjing, China). Four-week-old male C57BL/6 mice were obtained from the Animal Core Facility of Nanjing Medical University and housed under specific pathogen-free (SPF) conditions. To induce fibrotic changes, a single installation with 50 mg/kg SiO_2_ dissolved in 0.05 ml sterile saline or 0.05 ml sterile saline was injected into mice lungs. Mice were harvested after 7, 14, 28 days of treatment, and the lungs were collected and stored at -80 °C.

The adeno-associated virus 9 (AAV9) packed with pre-miR-30a or sh-circHIPK3 was designed by Gene Co., Ltd. (Shanghai, China). A total of 40 male C57BL/6 mice were used for this animal experiment. These mice were randomly divided into five groups (*n*=8 each group): control, SiO_2_ group, SiO_2_+ AAV-miR-30a-3p, SiO_2_ +AAV-sh-circHIPK3, and SiO_2_ +AAV-control. Mice were administered with a 50 μl AAV9 virus at a titer of 1 × 10^11^ v.g./ml per mouse intratracheally. After 3 weeks, mice were treated with 50 mg/kg SiO_2_ using the above method, and the control group was treated with 0.05 ml of sterile saline. Another 4 weeks later, all mice were sacrificed, and lungs from mice were isolated and stored at -80 °C immediately for further analysis.

### Histopathology

The fresh mouse lung tissues were collected and soaked in formalin solution overnight. After embedded in paraffin, lung tissues were sectioned into 5μm-thick slices. Then, lung tissues were used for H&E staining, Masson staining, and immunohistochemistry analysis of Col-1 expression, followed by scanning with Pannoramic Scanning Electron Microscope. The structure changes of mouse lungs were measured base on the degree of alveolar wall thickening, inflammatory lesions, collagen deposition, and cellular proliferation.

### Cell culture and treatment

MRC-5 and NIH/3T3 were purchased from the Chinese Academy of Sciences Cell Bank (Shanghai). For maintenance, cells were maintained in the appropriate medium (MEM for MRC-5, and DMEM for NIH/3T3 and mouse primary lung fibroblasts) supplemented with 10% fetal bovine serum (FBS), 100 U/ml penicillin, and 100 μg/ml streptomycin (Life Technologies/Gibco, Gaithersburg, MD) at 37 °C with 5% CO_2_. Fibroblasts (MRC-5 and mouse primary lung fibroblasts) were cultured in medium added with 5 ng/ml recombinant TGF-β1 (Peprotech) for 48 h to induce fibroblast activation. To inhibit glycolysis, MRC-5 cells were pretreated with 2-Deoxy-d-glucose (2-DG) at various concentrations (1, 3, 10 mM) for 1 hour, followed by TGF-β1 treatment for 48 hours.

### Cell transfection

CircHIPK3 siRNA, FOXK2 siRNA, control siRNA, miR-30a-3p mimic, miR-30a-3p inhibitor, control mimic, control inhibitor, circHIPK3 plasmid, and FOXK2 plasmid were designed and synthesized by GenePharm (Shanghai, China) Transfection was carried out using riboFECTCP Reagent (Ribobio, Guangzhou, China) according to the manufacturer's protocol. The sequences of CircHIPK3 siRNA and FOXK2 siRNA were shown as follows: circHIPK3, Forward primer, 5'- GUACUACAGGUAUGGCCUCTT-3', Reverse primer, 5'- GAGGCCAUACCUGUAGUACTT-3'; FOXK2, forward primer, 5'-GCGAGUUCGAGUAUCUGAUTT-3', Reverse primer, 5'- AUCAGAUACUCGAACUCGCTT -3'.

### RNase R and Actinomycin D treatment

For the RNase R treatment, 3 μg total RNA extracted from MRC-5 cells was incubated for 15 minutes with 3U/μg RNase R or control buffer (Epicentre, USA). Then, the expression of circHIPK3 and HIPK3 was measured by qRT-PCR or RT-PCR. To block transcription, 2 μM actinomycin-D (Glpbio) was added into the cell culture medium, and cells were collected at different time points to extract total RNA for qRT-PCR analysis.

### RNA isolation and Real-time PCR

Total RNA was isolated from mouse tissues and cells using Trizol reagent (TianGen) and reverse-transcribed into complementary DNA (cDNA) using HiScript® II Q RT SuperMix for qPCR Kit. MiRNAs were reverse-transcribed with specific primers using a HiScript® II Q Select RT SuperMix for qPCR Kit (Vazyme Biotech Co., Ltd., Nanjing, China). The qRT-PCR was carried out with an AceQ® qPCR SYBR® Green Master Mix kit (Vazyme Biotech Co., Ltd., Nanjing, China) on a LightCycelr ®480II machine following the manufacturer's instructions. GAPDH was used as an endogenous control for the detection of circHIPK3 and FOXK2, and miRNAs expression was normalized to U6 small nuclear RNA. All the primers in this study were shown in Supplementary Table 1.

### Western blot analysis

The culture cells were lysed in RIPA lysis buffer containing protease and phosphatase inhibitors (Beyotime, China), and the total protein of mouse tissues was extracted using T-PER Tissue protein Extraction Reagent (Thermo Scientific Pierce). The BCA Protein Assay kit (Beyotime) was used to quantify protein concentration. Protein samples were separated by 10% SDS-PAGE gel and transferred onto PVDF membranes (Millipore). Then, membranes were blocked with 5% fat-free milk and incubated with primary antibodies overnight at 4 °C. After washed with TBST for 15 minutes, the membranes were further incubated with secondary antibodies for 1 hour at room temperature. Subsequently, membranes were washed with TBST for 45 minutes and imaged immediately using ChemiDoc XRS + (Bio-Rad Laboratories).

Primary antibodies: antibody against Fibronectin (ab6328, Abcam), antibody against Collagen I (ab34710, Abcam), antibody against FOXK2(ab5298, Abcam), antibody against α-SMA (ab32575, Abcam), antibody against HK2 (22029-1-AP, Proteintech), antibody against PFKM (ab154804, Abcam), antibody against PDK1 (ab202468, Abcam), antibody against PKM2 (4503, Cell Signaling Technology), antibody against Foxk2 (A14245, Abclonal), and GAPDH (AC002, Abclonal).

### Dual-luciferase reporter gene assay

Putative wild-type (WT) and mutant (Mut) miR-30a-3p-binding sites in the 3ʹ-UTR of circHIPK3 or FOXK2 were cloned into a pmirGLO-Report luciferase vector (Generay Biotechnology, China). The reporter plasmid and miR-30a-3p mimic or inhibitor were co-transfected into MRC-5 cells using transfection reagent (RiboBio Co., Ltd., Guangzhou, China) according to the manufacture's instruction. After 24 h transfection, Luciferase activities were evaluated with the Dual-Luciferase Reporter Assay System kit (Beyotime, China).

### Immunofluorescence experiment

MRC-5 cells were fixed with 4% paraformaldehyde for 15 min. After washing three times with PBS, the cells were blocked with 5% BSA for 1 h at room temperature and incubated with primary antibody against α-SMA (ab32575, Abcam) or antibody against Collagen I (ab34710, Abcam) overnight at 4 °C. After washing with PBST, the cells were incubated with a Cy3-conjugated goat anti-rabbit or FITC conjugated goat anti-rabbit secondary antibody (Beyotime, China) for 1 h. The nuclear were then stained with DAPI. Fluorescent images were acquired under a fluorescence microscope (Zeiss, LSM700B, Germany).

### RNA immunoprecipitation (RIP)

RIP assays were carried out using the Magna RIP RNA-Binding Protein Immunoprecipitation Kit (Millipore, Billerica, MA, USA). 1 × 10^7^ MRC-5 or NIH/3T3 cells were harvested and resuspended in 200 μl of RIP Lysis Buffer combined with protease inhibitor cocktail and RNase inhibitor. The cell supernatants (200 μl) were incubated with 10 μg of anti-AGO2 antibody (ab32381, Abcam) or rabbit IgG-coated magnetic beads followed by 24 h of rotating at 4 °C. After treated with proteinase K and RNAse-free DNase I, immunoprecipitated RNA was extracted from the lysates. The abundance of circHIPK3, FOXK2, and miR-30a-3p was detected by qRT-PCR.

### Fluorescence *in situ* hybridization (FISH)

FISH assays were performed to observe the location of circHIPK3 and miR-30a-3p in MRC-5 cells. The RNA FISH probes against circHIPK3 or miR-30a-3p were designed and synthesized by GenePharma Co., Ltd. (Shanghai, China). After prehybridization at 55 °C for 2 h, cells were hybridized with a specific Cy3-labeled circHIPK3 probe and FAM-labeled miR-30a-3p probe at 37 °C overnight. After washed twice with PBS, cells were incubated with DAPI for nuclear staining. Then, cells were immediately photographed with a fluorescence microscope (Leica, Wetzlar, Germany).

### RNA pull-down

Biotin labeled-miRNAs and circHIPK3 probe were designed and synthesized by GenePharma Co., Ltd. (Shanghai, China) for RNA pull-down assay. 1 × 10^7^ MRC-5 cells were collected, and total RNA was extracted. The RNA complex was pulled down by incubating the cell lysates with streptavidin-coated magnetic beads on a rotator at 4 °C overnight. After the binding process, the beads were washed twice with the 1× wash buffer (Thermo Scientific) and incubated with the elution buffer (Thermo Scientific) for 45 min at 37 °C. The abundance of circHIPK3, FOXK2, and miRNAs in the supernatant was evaluated by RT-qPCR analysis.

### Cell cytoplasm/nucleus fraction isolation

The detection of cytoplasm/nucleus fraction percentage was performed using the Thermo PARIS^TM^ Kit (Thermo Fisher, USA) according to the manufacturer's protocol. Briefly, 2 × 10^6^ MRC-5 cells were collected and washed two times with PBS. Then, 300 μl Cell Fractionation Buffer was added to resuspend the cells. Centrifuge at low speed (500×g) at 4 °C for 5 minutes to separate the cytoplasm/nucleus fraction, and the extracted RNAs from the cytoplasm or the nucleus were obtained after further purification. The relative expression levels of circHIPK3, miR-30a-3p, nuclear control transcript (U6), and cytoplasmic control transcript (GAPDH) were measured using qRT-PCR or RT-PCR.

### Metabolic studies

Glucose consumption, lactate production, glycolytic rate, and ATP production were performed with kits according to the manufacturer's instructions. Glucose levels were determined by the use of a glucose assay kit (BioVision, Milpitas, CA, USA). Lactate in cell culture media was detected by Lactic Acid assay kit (Jiancheng, China, A019-2-1). A glycolysis assay kit (Abcam, ab197244) was used to measure the glycolytic rate, and an ATP Assay Kit (Beyotime, China, S0027) was used to detect the production of ATP.

### Statistical analysis

All experiments were repeated at least in triplicates and data were presented as mean ± SD. The comparisons between the two groups were performed using a two-tailed unpaired t-test for normally distributed data. Multiple group comparisons were performed using a one-way analysis of variance (ANOVA) with Dunnett's test. All statistical analysis was done using SPSS 17.0 and *P*‐values < 0.05 were considered significant.

## Results

### Glycolysis plays a crucial role in TGF-β1-induced pulmonary fibroblast activation

**Figure 1:**
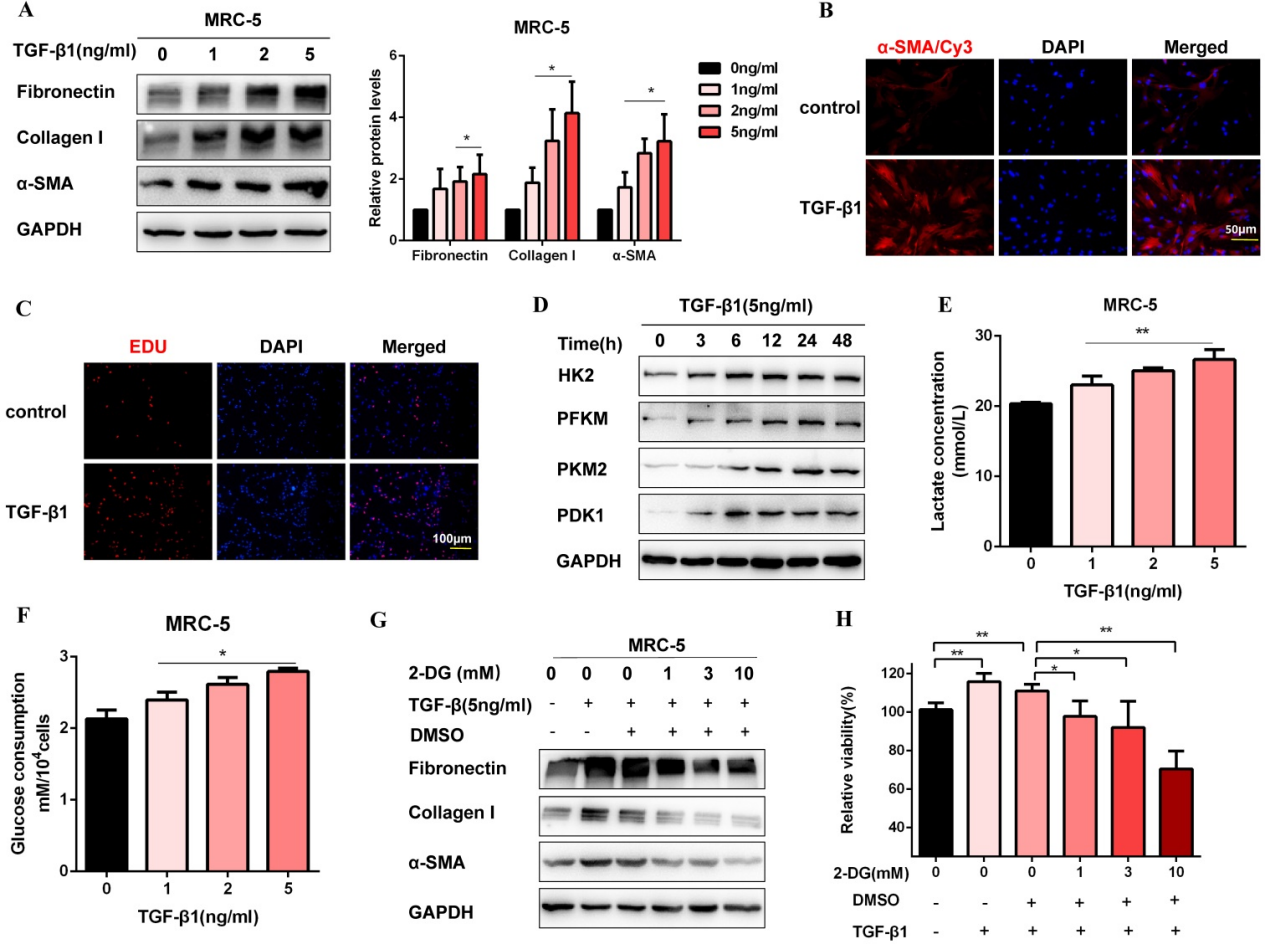
** Glycolysis plays a key role in TGF-β1-induced pulmonary fibroblast activation.** (A) Western blot and densitometric analysis of Fibronectin, Collagen I, and α-SMA in MRC-5 cells were treated with 0, 1, 2, 5 ng/ml TGF-β1 for 48h. (B) Immunofluorescence staining of α-SMA in MRC-5 cells for the control and TGF-β1 (5 ng/ml) treatment groups. Red represents α-SMA staining; blue represents nuclear DNA staining by DAPI. (C) DNA synthesis was assessed using EDU assay in MRC-5 cells for the control and TGF-β1 (5 ng/ml) treatment groups. Red, EDU; blue, nuclei. (D) Western blot detected levels of HK2, PFKM, PKM2, and PDK1 in MRC-5 cells were treated with 5 ng/ml TGF-β1 for 0h, 3h, 6h, 12h, 24h, 48h. (E-F) MCR-5 cells were treated with 0, 1, 2, 5 ng/ml TGF-β1 for 48 h. Lactate levels and glucose consumption were determined (*n* = 3), with *P < 0.05, **P < 0.01 vs. the control group. (G-H) MRC-5 cells were pretreated with 2-DG (1 mM, 3 mM, 10 mM) for 1 hour, followed by TGF-β1 treatment for 48 hours. Fibronectin, Collagen I, and α-SMA expression were determined by western blot, and cell viability was detected by MTT assays (*n* = 3), **P < 0.01.

Firstly, we established a fibroblast activation cell model induced by TGF-β1. To identify TGF-β1 induced fibroblast activation, we performed western blot for MRC-5 cells treated with TGF-β1 (0, 1, 2, 5 ng/ml) for 48 h. As expected, profibrotic factors (Fibronectin, Collagen I, and α-SMA) were significantly increased in a dosed manner, and treatment with 5 ng/ml TGF-β1 resulted in a saturation level (Figure 1A). Therefore, we chose 5 ng/ml TGF-β1 to treat cells for 48 h in the subsequent experiments. Immunofluorescence staining of α-SMA was also up-regulated after TGF-β1 treatment, which was accompanied by the increase of proliferation activity (Figure 1B, Figure 1C, Figure S1A, and Figure S1B). Moreover, the protein expression of glycolytic enzymes significantly increased in TGF-β1-stimulated MRC-5 cells, including hexokinase-II (HK2), phosphofructokinase, muscle (PFKM), pyruvate kinase M2 (PKM2), and pyruvate dehydrogenase kinase 1 (PDK1) (Figure 1D and Figure S1C). Besides, the glycolytic alteration was also supported by the increased lactate production and glucose consumption after TGF-β1 treatment (Figure 1E and Figure 1F). However, 2-Deoxy-d-glucose(2-DG), an inhibitor of glycolysis, inhibited fibrosis markers production and cell proliferation induced by TGF-β1(Figure 1G, Figure 1H, and Figure S1D). Altogether, the data suggested that enhanced glycolysis contributed to fibroblast activation.

### CircHIPK3 is involved in TGF-β1-derived fibroblast activation and proliferation

**Figure 2:**
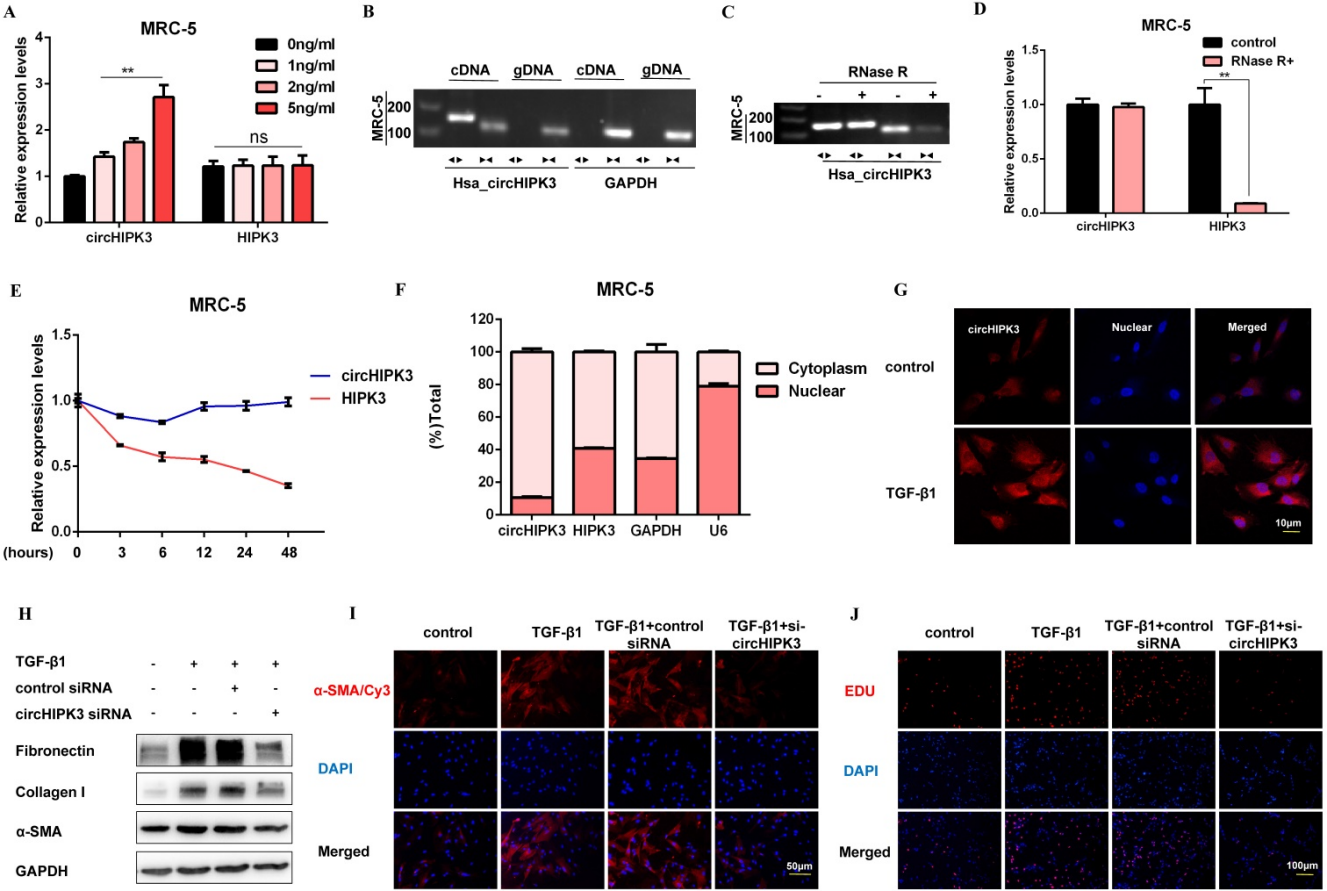
** CircHIPK3 is involved in TGF-β1-derived fibroblast activation and proliferation.** (A) CircHIPK3 and HIPK3 mRNA expression were determined by qRT-PCR in MRC-5 cells treated with 0, 1, 2, 5 ng/ml TGF-β1 for 48h (*n* = 3), with **P < 0.01 vs. the control group. (B) RT-PCR validated the existence of circHIPK3 in MRC-5 cell lines. CircHIPK3 was amplified by divergent primers in cDNA but not gDNA. GAPDH was used as a negative control. (C-D) The expression of circHIPK3 and HIPK3 mRNA in MRC-5 was detected by RT-PCR or qRT-PCR in the presence or absence of RNase R. (E) The abundance of circHIPK3 and HIPK3 mRNA was assessed by qRT-PCR MRC-5 cells treated with Actinomycin D (2μM) at the indicated time points. (F) Expression of circHIPK3 and HIPK3 in nuclear and cytoplasm of MRC-5 were measured via qRT-PCR analysis. (G) Fluorescence *in situ* hybridization (FISH) assay was conducted to determine the subcellular localization and expression of circHIPK3 in control and TGF-β1-treated groups. (H) Western blot analysis of Fibronectin, Collagen I, and α-SMA in MRC-5 cells transfected with circHIPK3 siRNA or its negative control then treated with 5ng/ml TGF-β1 for 48h. (I) Immunofluorescence staining of α-SMA in MRC-5 cells for the indicated groups. Red represents α-SMA staining; blue represents nuclear DNA staining by DAPI. (J) EDU assays of MRC-5 cells transfected with control or circHIPK3 siRNA were performed to evaluate cell proliferative ability.

Following TGF-β1 treatment, circHIPK3 was sufficiently up-regulated in a dose-dependent manner, while no significant changes of HIPK3 mRNA were observed (Figure 2A). To confirm circHIPK3 was the result of trans-splicing rather than genomic rearrangements, cDNA and gDNA were extracted separately from MRC-5 cells and subjected to amplify circHIPK3 and HIPK3 mRNA. As expected, circHIPK3 could be detected only in cDNA, as no products were detected in the extracted gDNA (Figure 2B). Following RNase R treatment, the levels of HIPK3 mRNA decreased sharply, but circHIPK3 showed strong resistance to digestion by RNase R (Figure 2C and Figure 2D). Accordingly, circHIPK3 was more stable than linear HIPK3 regarding Actinomycin D treatment (Figure 2E). These data indicated that circHIPK3 harbors a loop structure. Nuclear separation experiments showed that circHIPK3 was enriched in the cytoplasm (Figure 2F). Consistently, fluorescence *in situ* hybridization (FISH) assay also suggested that circHIPK3 predominately localized in the cytoplasm, and robust induction of circHIPK3 was observed after TGF-β1 treatment (Figure 2G).

To investigate whether circHIPK3 is required for fibroblast activation, we performed loss-of-function experiments using small-interfering RNAs (siRNAs). We designed a siRNA-targeted circHIPK3, which could significantly reduce the expression of circHIPK3 but not HIPK3 (Figure S2A). As expected, circHIPK3 knockdown substantially exerted potent antifibrotic effects by inhibiting the expression of profibrotic proteins and staining of α-SMA in MRC-5 cells (Figure 2H, Figure 2I, Figure S2B, and Figure S2C). Also, EDU assays showed that loss of circHIPK3 significantly decreased the proliferation activity of MRC-5 cells (Figure 2J and Figure S2D). Notably, silencing circHIPK3 inhibited lactate production and glucose consumption, indicating that circHIPK3 was also associated with glycolysis in fibroblasts (Figure S2E and Figure S2F). These results collectively indicated a major role of circHIPK3 in fibroblast activation, and the fibroblast phenotype can be rescued after circHIPK3 knockdown.

### CircHIPK3 acts as a sponge for miR-30a-3p in lung fibroblasts

**Figure 3:**
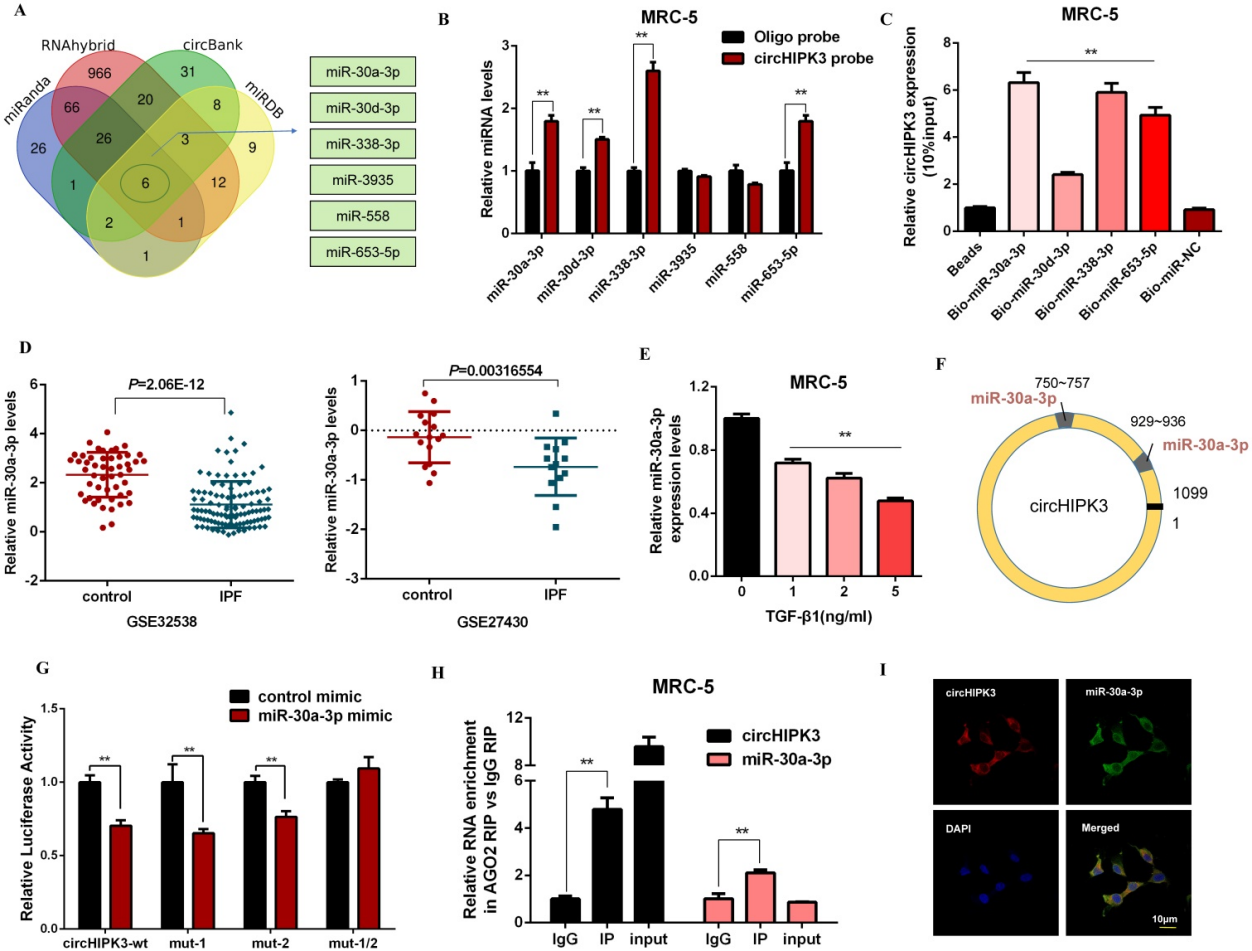
** CircHIPK3 acts as a sponge for miR-30a-3p in lung fibroblasts.** (A) Schematic illustration exhibiting overlapping of the target miRNAs of circHIPK3 predicted by miRanda, miRDB, circBank, and RNAhybrid. (B) The relative levels of miRNAs in MRC-5 cells were pulled down by a circHIPK3 probe. (C) The relative levels of circHIPK3 in MRC-5 cells were pulled down by biotinylated miR-30a-3p, miR-30d-3p, miR-338-3p and miR-653-5p (*n* = 3), with **P < 0.01 vs. the Bio-miR-NC group. (D) The miR-30a-3p was down-regulated in IPF patients based on the microarray dataset GSE32538 and GSE27430. (E) MiR-30a-3p expression were determined by qRT-PCR in MRC-5 cells treated with 0, 1, 2, 5 ng/ml TGF-β1 for 48h (*n* = 3), with **P < 0.01 vs. control group. (F) Schematic of putative binding sites of miR-30a-3p on circHIPK3 transcript. (G) MRC-5 were co-transfected with LUC-circHIPK3-wt, LUC-circHIPK3-mut-1, LUC-circHIPK3-mut-2, or LUC-circHIPK3-mut-1/2 with miR-30a-3p mimics or scrambled mimics. Luciferase activity was detected 24 h after transfection (*n* = 3), **P < 0.01. (H) RIP assays for circHIPK3 and miR-30a-3p levels in MRC-5 cells were detected by qRT-PCR analysis (*n* = 3), **P < 0.01. (I) Colocalization between miR-30a-3p and circHIPK3 was observed via FISH assays in MRC-5 cells. CircHIPK3 probes were labeled with Alexa Fluor 555. MiR-30a-3p probes were labeled with Alexa Fluor 488. Nuclei were stained with DAPI.

It has been reported that circRNAs act as miRNA sponges and subsequently abolish the corresponding miRNA function. Given that circHIPK3 predominantly localized in the cytoplasm and exhibited stability, we explored whether circHIPK3 influenced fibroblast activation by sponging miRNAs. Then, four databases (miRanda, miRDB, circBank, and RNAhybrid) were used to predict the potential target miRNAs of circHIPK3, and among which 6 miRNAs were selected from the overlap between these databases (Figure 3A). Then, we performed a pull-down assay with a biotinylated circHIPK3 probe and selected 4 possible miRNAs with significantly enhanced fold-changes for circHIPK3 capture (Figure 3B). Furthermore, greater enrichment of endogenous circHIPK3 was observed in the biotinylated miR-30a-3p-captured fraction in comparison with the negative control (Figure 3C). Interestingly, we also found miR-30a-3p was significantly down-regulated in IPF patients based on the microarray datasets GSE32538 and GSE27430 (Figure 3D). Moreover, the expression of miR-30a-3p was negatively regulated by TGF-β1 and circHIPK3 (Figure 3E, Figure S3A, and Figure S3B).

According to the bioinformatics analysis, circHIPK3 has two putative binding sites for miR-30a-3p (Figure 3F). Compared to the control group, miR-30a-3p mimic significantly reduced the luciferase reporter activities of circHIPK3-wt, circHIPK3-1-mutant, circHIPK3-2-mutant vectors, but did not affect the luciferase activities of the mutant vector, indicating that miR-30a-3p could directly bind to these two sites in circHIPK3 (Figure 3G). Then, nuclear separation experiments showed that miR-30a-3p was located in both nucleus and cytoplasm (Figure S3C). Next, a RIP assay with AGO2 antibody suggested that endogenous circHIPK3 and miR-30a-3p were predominantly enriched in the AGO2 antibody group, which further demonstrated the interaction between miR-30a-3p and circHIPK3 (Figure 3H). Besides, co-localization between circHIPK3 and miR-30a-3p was also observed in the cytoplasm by FISH analysis (Figure 3I). Overall, our data suggest circHIPK3 could act as a sponge of miR-30a-3p. Interestingly, we observed that miR-30a-3p could negatively regulate the expression of circHIPK3, indicating that there might be a mutual regulatory relationship between miR-30a-3p and circHIPK3 (Figure S3D and Figure S3E).

### MiR-30a-3p mediates the function of circHIPK3 to regulate fibroblast activation

**Figure 4:**
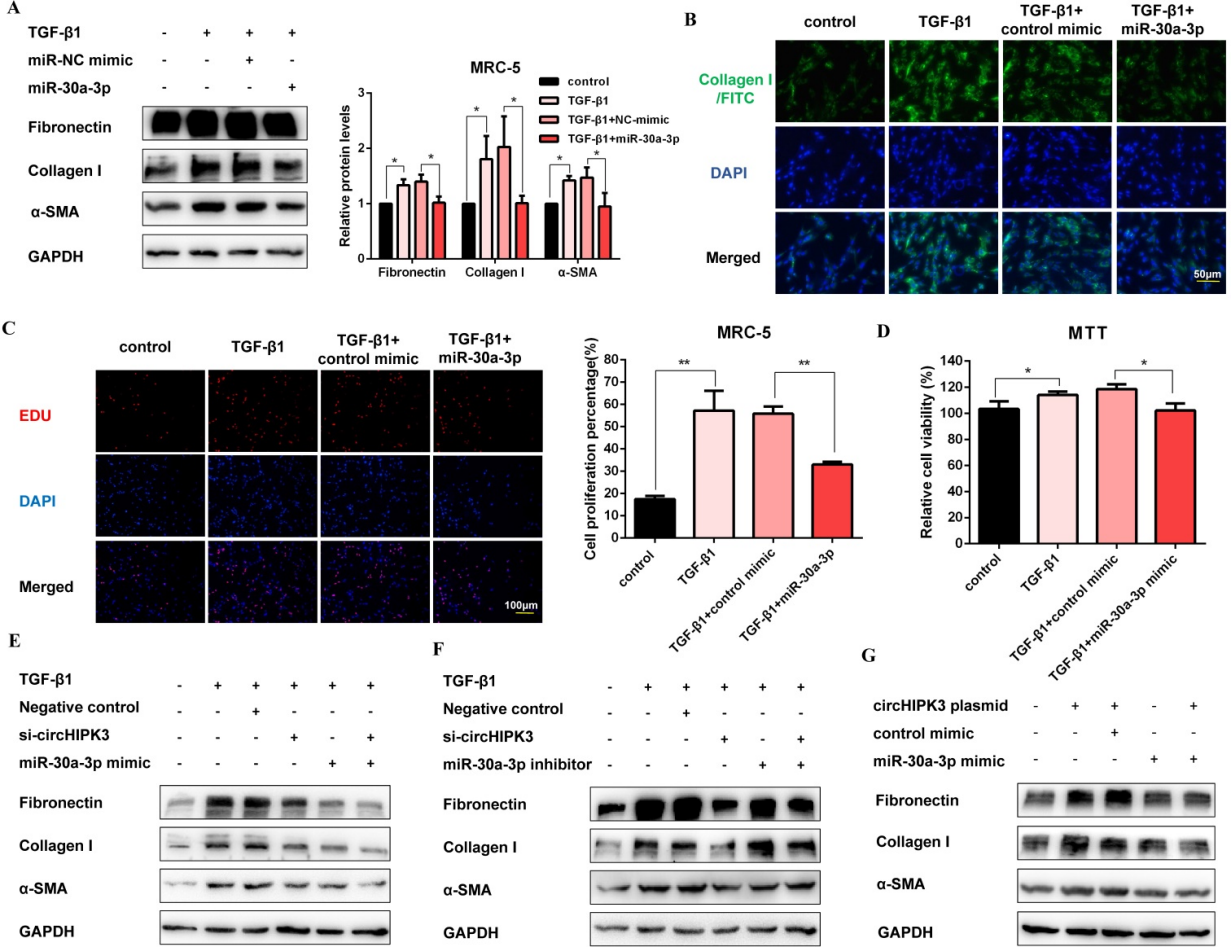
** MiR-30a-3p mediates the function of circHIPK3 to regulate fibroblast activation.** (A) Western blot and densitometric analysis of Fibronectin, Collagen I, and α-SMA in MRC-5 cells transfected with miR-30a-3p or control mimic then treated with 5ng/ml TGF-β1 for 48h (*n* = 3), *P < 0.05. (B) Immunofluorescence staining of Collagen I in MRC-5 cells for the indicated groups. Green represents Collagen I staining; blue represents nuclear DNA staining by DAPI. (C-D) EDU and MTT assays were performed to evaluate cell proliferative ability in MRC-5 cells (*n* = 3), *P < 0.05, **P < 0.01. (E-G) Western blot analysis of the protein expression of Fibronectin, Collagen I, and α-SMA in treated MRC-5 cells for the indicated groups.

To explore the function of miR-30a-3p during the fibroblast activation, we elevated the level of miR-30a-3p by mimic transfection (Figure S4A). As expected, western blot showed that miR-30a-3p overexpression decreased the production of fibrosis markers (Figure 4A). Moreover, staining of Collagen I in MRC-5 cells further confirmed the anti-fibrotic effects of miR-30a-3p. (Figure 4B and Figure S4B). Then, EDU and MTT assays also revealed that miR-30a-3p overexpression inhibited fibroblast proliferation activity induced by TGF-β1 (Figure 4C and Figure 4D). As shown in Figure 4E and Figure S4C, both circHIPK3 knockdown and miR-30a-3p overexpression could block TGF-β1-induced fibroblast activation. Moreover, the miR-30a-3p inhibitor was able to partly rescue the loss of Fibronectin, Collagen I, and α-SMA caused by the circHIPK3 knockdown (Figure 4F and Figure S4D). Besides, over-expressed circHIPK3 could induce fibroblast activation, whereas the effects were reversed by miR-30a-3p mimic (Figure 4G and Figure S4E). These results revealed that miR-30a-3p mediated the function of circHIPK3 to regulate fibroblast activation.

### FOXK2 is a functional target of miR-30a-3p and exerts profibrotic effects by regulating glycolysis

**Figure 5:**
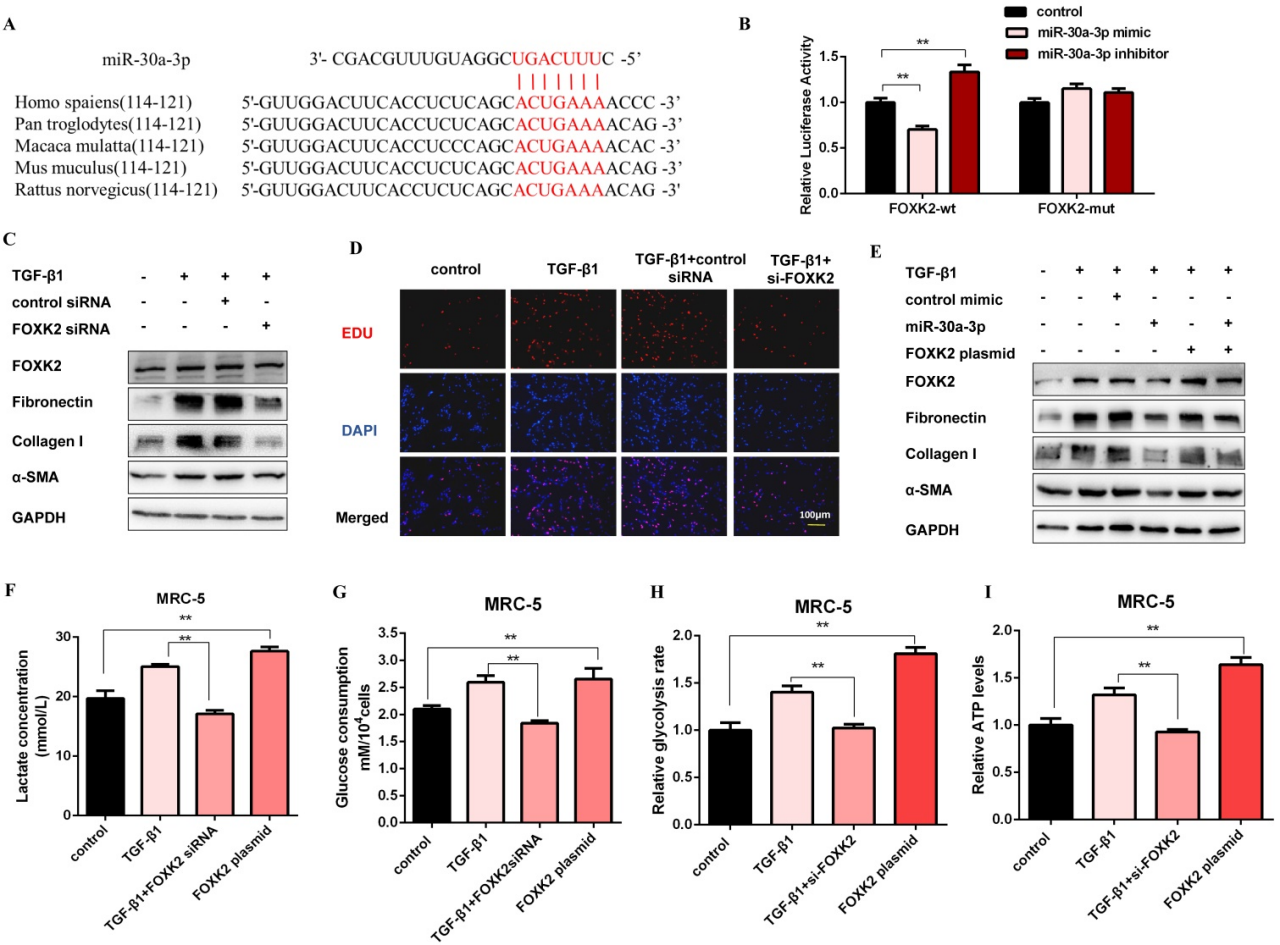
** FOXK2 is a functional target of miR-30a-3p and exerts profibrotic effects by regulating glycolysis.** (A) Schematic diagram of conserved target sites of miR-30a-3p in the 3'UTR of several species FOXK2 mRNA. (B) Luciferase activities of Luc-FOXK2-wild or Luc-FOXK2-mutant in MRC-5 cells co-transfected with miR-30a-3p mimic, miR-30a-3p inhibitor, or N.C. were determined by a luciferase reporter assay (*n* = 3), **P < 0.01. (C) Western blot detected levels of FOXK2, Fibronectin, Collagen I, and α-SMA in MRC-5 cells transfected with FOXK2 siRNA or its negative control then treated with 5 ng/ml TGF-β1 for 48 h. (D) Cell proliferation was detected using EDU assay. Red, EDU; blue, nuclei. Scale bar, 100μm. (E) FOXK2, Fibronectin, Collagen I, and α-SMA expression levels were detected by western blot analysis. (F-I) Lactate concentration, glucose consumption, glycolytic rate, and ATP concentration were detected in MRC-5 cells for the indicated groups (*n* = 3), **P < 0.01.

Next, we elucidated the probable target genes of miR-30a-3p via bioinformatics analysis and deemed FOXK2 as a potential target of it. One putative miR-30a-3p-binding site within the 3'-untranslated region (3'-UTR) of human FOXK2 was observed, and the binding site is also conserved in several species (Figure 5A). Then, we detected the mRNA and protein expression levels of FOXK2 in MRC-5 cells with miR-30a-3p overexpression or knockdown. As shown in Figure S5A and Figure S5B, FOXK2 was negatively correlated with the expression of miR-30a-3p at protein but not mRNA levels. Then, dual-luciferase reporter assays confirmed the direct interaction between miR-30a-3p and FOXK2 (Figure 5B). This observation was further verified by the finding that endogenous FOXK2 mRNA could be pulled down by biotin-labeled miR-30a-3p (Figure S5C). TGF-β1 effectively increased the expression of FOXK2 and profibrotic factors (Fibronectin, Collagen I, and α-SMA), however, FOXK2 knockdown in MRC-5 cells strikingly inhibited the production of these proteins (Figure 5C and Figure S5D). As indicated by the EDU assays, FOXK2 knockdown also reduced the proliferation of MRC-5 induced by TGF-β1 (Figure 5D and Figure S5E). Besides, overexpression of FOXK2 rescued the decreased fibrosis markers caused by the transfection of miR-30a-3p mimic (Figure 5E and Figure S5F). Taken together, these data indicated that miR-30a-3p exerted anti-fibrosis functions, at least partially, by directly targeting FOXK2.

To investigate the role of FOXK2 in glycolysis, the expression of glycolytic enzymes was measured by western blot. As shown in Figure S5G and Figure S5H, the protein levels of HK2, PFKM, PKM2, and PDK1 were negatively correlated with the expression of FOXK2. Accordingly, lactate level, glucose uptake, glycolytic rate, and ATP production were all decreased after FOXK2 knockdown but increased in FOXK2-overexpressed MRC-5 cells (Figure 5F-5I). Moreover, FOXK2 overexpression also induced the production of fibrosis markers, whereas its effects were reversed by 2-DG (Figure S5I). These data suggested that FOXK2 could promote fibroblast activation by facilitating glycolysis.

### CircHIPK3 and miR-30a-3p are dysregulated during pulmonary fibrogenesis

**Figure 6:**
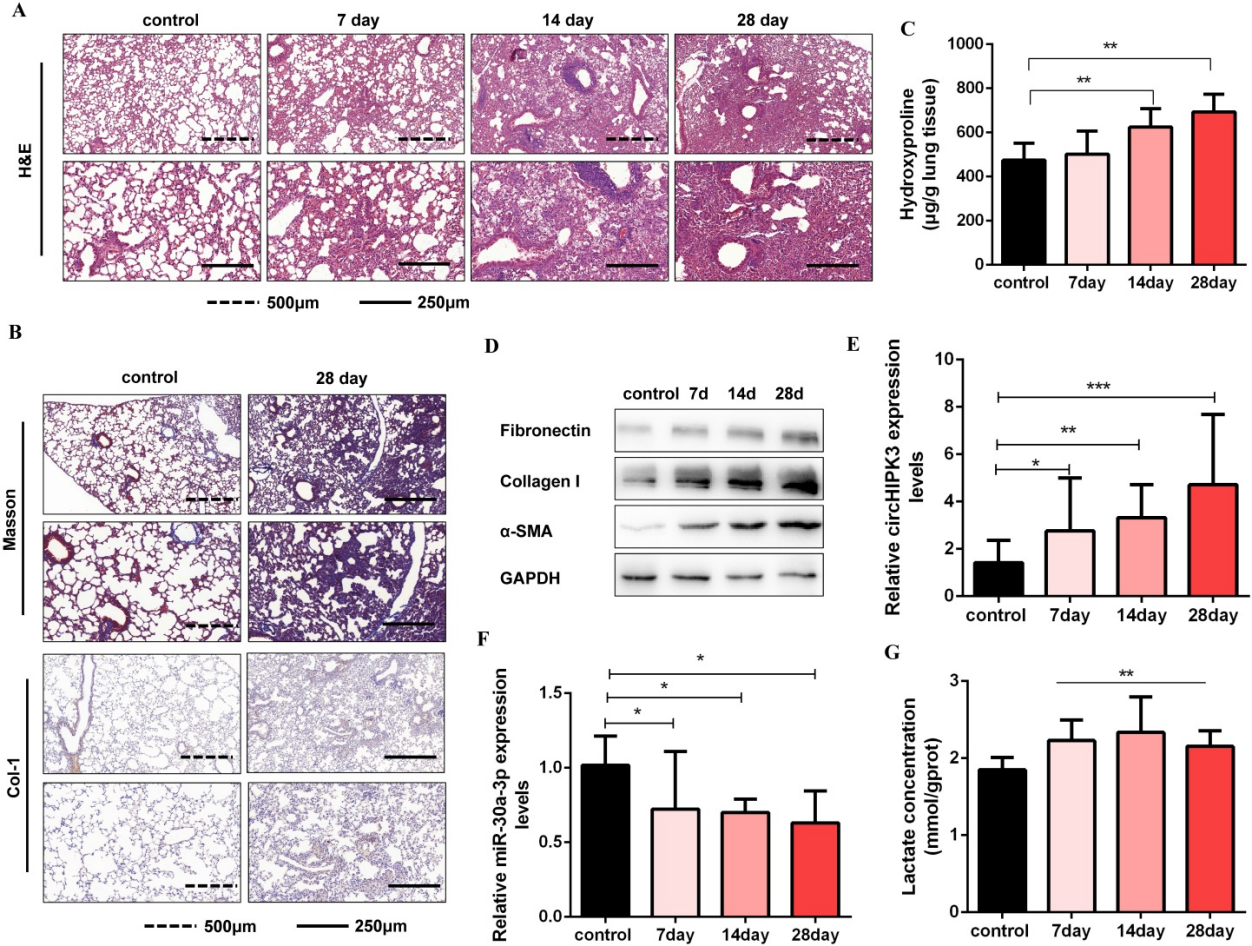
** CircHIPK3 and miR-30a-3p are dysregulated during pulmonary fibrogenesis.** (A) The C57BL/6 mice were sacrificed on days 7, 14, and 28 after intratracheal instillation of silica suspended saline and saline. Histological changes in lung tissues were observed by hematoxylin and eosin (H&E) staining. (B) Masson staining and IHC staining assays were performed to measure fibrotic lesions and Col-1 expression. (C) Hydroxyproline content of the lung tissues was used to assess the degree of collagen deposition. (D) Western blot and densitometric analysis of the protein expression of Fibronectin, Collagen I, and α-SMA in mouse lung tissues. GAPDH was used as an internal loading control. (E-F) qRT-PCR analysis of circHIPK3 and miR-30a-3p expression in mouse fibrotic lung tissue on days 7, 14, and 28. (G) Lactate concentration was detected in lung tissues exposed to silica for 7, 14, and 28 days (*n* = 6), with **P < 0.01 vs. the control group.

Circbase retrieval shows that one of the isoforms of circHIPK3 is highly conserved between humans (chr11:33307958-33309057, hsa_circ_0000284) and mouse genome (chr2:104310905-104312004, mmu_circ_0001052). Then, we found that miR-30a-3p had a complementary binding site with circHIPK3 (mmu_circ_0001052) and further confirmed the interaction between them. Consistent with the results in MRC-5 cells, we identified circHIPK3 as a determinant of mouse primary lung fibroblasts activation and partially ascribed its profibrotic effects to regulate miR-30a-3p/Foxk2 axis (Figure S6). To further verify the role of circHIPK3 and miR-30a-3p in pulmonary fibrosis *in vivo*, a silica-induced pulmonary fibrosis mouse model was established via the intratracheal administration of silica suspension. The SiO_2_ concentration (50mg/kg) was determined based on the literature review and pre-experimental results. We have observed that this concentration of SiO_2_ successfully induces pulmonary fibrosis in mice but does not affect mice's survival. As shown by H&E staining, the normal alveolar architecture was destroyed and mature fibrotic nodules formed after silica exposure for 28days (Figure 6A). Besides, Masson's trichrome staining and IHC staining to Collagen I indicated the excessive ECM deposition in the mouse lungs on day 28 (Figure 6B), and the results were further confirmed by hydroxyproline analysis (Figure 6C). Silica injury also increased the expression of several profibrotic mediators, including α-SMA, Collagen I, and Fibronectin (Figure 6D and Figure S7A). Next, we detected circHIPK3 and miR-30a-3p expression levels in this model and observed circHIPK3 was up-regulated, while miR-30a-3p was markedly down-regulated in a time-dependent manner (Figure 6E and Figure 6F). Besides, the lactate levels and the protein expression of Foxk2 and glycolytic enzymes (HK2, PFKM, PKM2, and PDK1) were also upregulated after silica exposure (Figure 6G and Figure S7B). These findings indicated that circHIPK3 and miR-30a-3p could have the potential function of regulating pulmonary fibrosis.

### CircHIPK3 and miR-30a-3p regulate silica-induced pulmonary fibrosis *in vivo*

**Figure 7:**
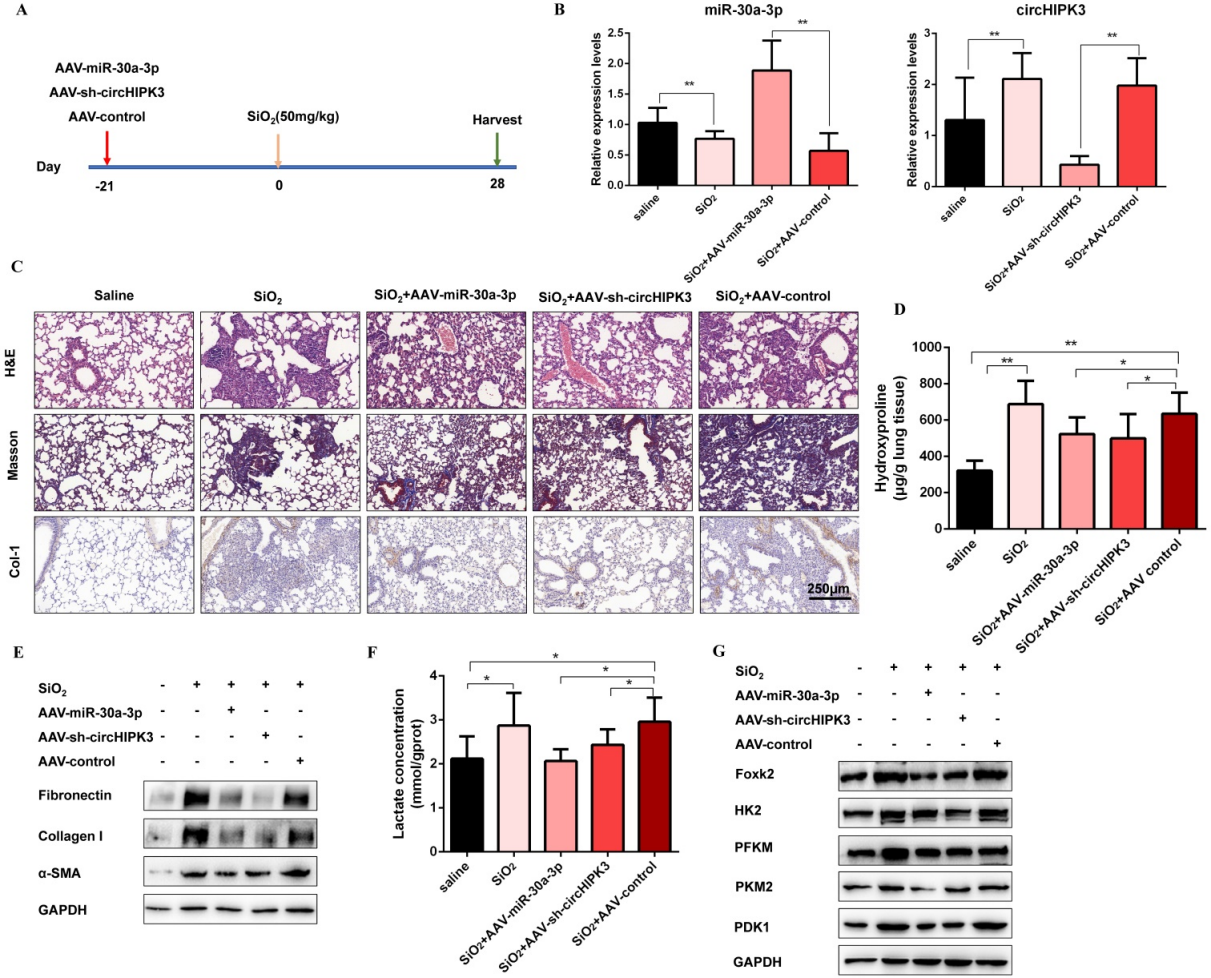
** CircHIPK3 and miR-30a-3p regulate silica-induced pulmonary fibrosis *in vivo*.** (A) Strategy for circHIPK3 knockdown or miR-30a-3p overexpression or in bleomycin silica-induced pulmonary fibrosis mouse model. (B) MiR-30a-3p and circHIPK3 expression were measured by qRT-PCR in the treated mouse lung tissues for the indicated groups. (C) H&E staining, Masson staining, and IHC staining of Collagen I were performed to measure the severity of the lung fibrosis. (D) MiR-30a-3p overexpression or circHIPK3 silence attenuated the hydroxyproline content in the lungs of mice treated with SiO_2_. (E) The protein expression of Fibronectin, Collagen I, and α-SMA in mouse lung tissues treated was determined by western blot. GAPDH was used as an internal loading control. (F) circHIPK3 silence or miR-30a-3p overexpression decreased the lactate levels in the lungs of mice treated with SiO_2_. (G) Foxk2, HK2, PFKM, PKM2, and PDK1 were detected by western blot analysis. GAPDH was used as an internal loading control.

To confirm the role of circHIPK3 and miR-30a-3p during pulmonary fibrosis progression, we designed adeno-associated viral (AAV) shRNAs for circHIPK3 silencing and adeno-associated viral (AAV) pre-miR-30a for miR-30a-3p overexpression (Figure 7A). Then, lung tissues were harvested, and qRT-PCR was performed to detect the expression levels of circHIPK3 and miR-30a-3p. As expected, AAV-pre-miR-30a intratracheal injection significantly elevated miR-30a-3p levels, and AAV-sh-circHIPK3 reduced lung circHIPK3 expression (Figure 7B). Histologically, extensive tissue fibrosis was observed after silica injury, whereas both circHIPK3 silencing and miR-30a-3p overexpression exceedingly attenuated pulmonary fibrosis (Figure 7C). In accordance, the data were further confirmed by hydroxyproline analysis (Figure 7D). Besides, western blot analysis showed that miR-30a-3p overexpression or circHIPK3 silence could decrease the expression of fibrosis markers (Fibronectin, Collagen I, and α-SMA) at protein levels (Figure 7E and Figure S8A). Compared to the silica treatment group, the lactate content and the protein levels of Foxk2, HK2, PFKM, PKM2, and PDK1 were also decreased in miR-30a-3p overexpression and circHIPK3 knockdown groups (Figure 7E and Figure S8B). Collectively, these data showed the regulatory function of circHIPK3 and miR-30a-3p in silica-induced pulmonary fibrosis.

## Discussion

Pulmonary fibrosis is the outcome of numerous interstitial lung diseases, many of which have a dismal prognosis, however, existing treatments are of limited benefit 23. Fibroblast activation is a central event that contributes to fibrosis progression and represents a major barrier to effective therapy 24. Understanding the molecular bases of abnormal fibroblast activation is essential to define novel antifibrotic therapy targets. In this study, we established a fibroblast activation cell model and a mouse silicosis model to evaluate the underlying molecular mechanisms of pulmonary fibrosis. Since no significant effects of silica on fibroblast activation were observed in our earlier studies, TGF-β1 was used to induce fibroblast activation instead of silica.

Over the past decades, circRNAs have aroused increasing attention since they are discovered as post-transcriptional modulators for gene expression. CircRNAs are dysregulated in many fibrosis diseases, such as cardiac fibrosis, renal fibrosis, liver fibrosis, and lung fibrosis 21, 25-27. However, to date, their roles in glucose metabolism reprogramming have not been reported. In this study, we focused on circHIPK3, a circRNA derived from Exon2 of the HIPK3 gene, which has been reported to be commonly expressed in multiple tissues and enriched in the brain, lung, heart, and colon 28. Several studies have suggested that circHIPK3 is dysregulated in numerous cancer diseases and contributes to cell proliferation 29, 30. Here, we observed TGF-β1 effectively increased circHIPK3 expression in lung fibroblasts, and circHIPK3 knockdown blocked fibroblast activation and proliferation. Compared with the linear HIPK3, circHIPK3 was more stable and was predominantly localized in the cytoplasm. Interestingly, circHIPK3 expression was positively associated with lactate production and glucose consumption, indicating that circHIPK3 might be involved in the process of fibroblast activation by regulating glycolysis.

One of the most popular function models for circRNAs is that circRNAs function as ceRNA to sponge miRNAs, therefore competing with a linear target for the binding of the RNA-induced silencing complex (RISC). When the circRNA is expressed, the miRNA will guide the RISC to bind the circRNA, ultimately causing the de-repression of the mRNA. AGO2, a critical component of the RISC, has been demonstrated to play a key role in mediating the interaction between circRNAs and miRNAs 31, 32. By bioinformatics analysis, we screened 6 miRNAs predicted as targets of circHIPK3 and confirmed that miR-30a-3p was capable of binding to it by luciferase and RNA pull-down assays. Besides, endogenous circHIPK3 and miR-30a-3p were predominantly enriched in the AGO2 antibody group, which further supported that circHIPK3 could interact and binds with miR-30a-3p as a ceRNA. MiR-30a-3p has been demonstrated to regulates cell proliferation in multiple cancers, such as gastric cancer, liver cancer, and lung cancer 33-35. However, its role in pulmonary fibrosis has not been reported. On the contrary, the expression of miR-30a-3p was down-regulated by TGF-β1 in lung fibroblasts as well as in fibrotic mouse lung tissues induced by silica. Mechanistically, circHIPK3 could sponge endogenous miR-30a-3p and decrease its expression. Moreover, miR-30a-3p overexpression blocked the effects of TGF-β1 and circHIPK3 on fibroblast activation, which was further confirmed that miR-30a-3p was a downstream effector of circHIPK3.

Next, we explored the potential targets of miR-30a-3p and confirmed FOXK2 as a functional target of miR-30a-3p. The protein expression of FOXK2 was highly in fibroblasts after TGF-β1 treatment, and a negative relationship was observed between miR-30a-3p and protein expression of FOXK2. Luciferase reporter assays and RNA pull-down assays further confirmed FOXK2 was a direct target of miR-30a-3p. FOXK2 is a member of the FOX family and plays many broad and distinct roles in cell proliferation, DNA damage, metabolism, and cancer progression 36-39. Recent studies have shown that FOXK2 is involved in aerobic glycolysis regulation as a key transcription factor, however, the relationship between FOXK2 and pulmonary fibrosis is still unknown. Our data suggested that TGF-β1, a fibroblast activation inducer, induced FOXK2 expression and glycolysis. FOXK2 knockdown abrogated the increase of glycolysis and fibrosis markers levels induced by TGF-β1, and conversely, the overexpression of FOXK2 overcame this increase. Besides, plasmid-medicated FOXK2 overexpression promoted fibroblast activation, whereas its effects were reversed by 2-DG. Collectively, in this study, we firstly demonstrated that FOXK2 is a driver of fibroblast activation and ascribed its profibrotic effects to glycolysis reprogramming regulation via increasing the expression of several glycolytic enzymes.

Although the ceRNA regulatory network is an effective mechanism of circRNA, we cannot exclude the possibility that circHIPK3 promotes fibrogenesis via other molecular mechanisms. Moreover, TGF-β1 was observed to enhance circHIPK3 expression, but few reports are on the underlying molecular mechanism between TGF-β1 and circHIPK3. Remarkably, our results showed that miR-30a-3p also negatively regulated circHIPK3 expression. Data from previous studies on nucleus miRNAs uncover their important functions in the RNA splice or transcription 40. Several nucleus miRNAs have been reported to mediate the cleavage of circRNAs in an AGO2 dependent manner, and we found that miR-30a-3p was located in the nucleus as well as cytoplasm, indicating that miR-30a-3p may be involved in the modulation of circHIPK3 expression via the medication of its cleavage 41, 42. However, additional studies are needed to elucidate the interactions between circHIPK3 and miRNA in pulmonary fibrosis.

In conclusion, our study revealed a novel circHIPK3-mediated mechanism underlying the pathology of pulmonary fibrosis. Under pathological conditions, TGF-β1 could trigger the expression of circHIPK3 in fibroblasts, consequently enhancing FOXK2 expression via sponging miR-30a-3p. These events are collectively responsible for the induction of fibroblast glycolysis and activation as well as silica-induced pulmonary fibrosis. We thus anticipate that strategies surrounding circHIPK3/miR-30a-3p/FOXK2 pathway targeting may represent a new effective therapeutic option to treat pulmonary fibrosis and other fibrotic diseases.

## Supplementary Material

Supplementary figures and tables.

## Abbreviations

circRNA: circular RNA
miRNA: microRNA
ceRNA: competing endogenous RNA
TGF-β1: transforming growth factor β1
HK2: Hexokinase-II
PFKM: Phosphofructokinase, muscle
PKM2: Pyruvate kinase M2
PDK1: Pyruvate dehydrogenase kinase 1
2-DG: 2-Deoxy-d-glucose
qRT-PCR: real-time quantitative polymerase chain reaction
RIP: RNA immunoprecipitation
siRNA: small interfering RNA
FMT: fibroblast-to-myofibroblast transition
FOXK2: forkhead box (FOX) K2
cDNA: complementary DNA
gDNA: genomic DNA
H&E staining: hematoxylin & eosin staining
IHC: Immunohistochemical analysis
3'-UTR: 3'-untranslated region
