# Supplementary material for: CircHIPK3 regulates pulmonary fibrosis by facilitating glycolysis in miR-30a-3p/FOXK2-dependent manner
Source: Int J Biol Sci. 2021 Jun 4;17(9):2294–307. doi: 10.7150/ijbs.57915 (PMC8241722; doi:10.7150/ijbs.57915)
Supplement: Supplementary file 1 — Supplementary figures and tables. [file ijbsv17p2294s1.pdf]

Supplementary information to:

CircHIPK3 regulates pulmonary fibrosis by facilitating glycolysis in miR-30a-3p/FOXK2-dependent manner

Qi Xu[1], Demin Cheng[1], Guanru Li [1], Yi Liu, Ping Li, Wenqing Sun, Dongyu Ma, Chunhui Ni[*]

Center for Global Health, Key Laboratory of Modern Toxicology of Ministry of Education, Department of Occupational Medical and Environmental Health, School of Public Health, Nanjing Medical University, Nanjing 211166, China

*Corresponding author: Chunhui Ni, Center for Global Health, Key Laboratory of Modern Toxicology of Ministry of Education, Department of Occupational Medical and Environmental Health, School of Public Health, Nanjing Medical University, Nanjing 211166, China. E-mail: chni@njmu.edu.cn(Ch.-H. Ni),chninjmu@126.com(Ch.-H. Ni)

[1]These authors contributed equally to this work and should be considered co-first authors.

Supplementary Table 1

Gene Name	Forward primer (5'-3')	Reverse primer (5'-3')
circHIPK3（Homo）	TATGTTGGTGGATCCTGTTCGGC	TGGTGGGTAGACCAAGACTTGTGA
HIPK3(Homo)	GACCTGAGGAGATCAAGCCG	ATTGGGGCCCATTCCTGAC
circHIPK3(Mus)	GGATCGGCCAGTCATGTATC	ACCGCTTGGCTCTACTTTGA
HIPK3(Mus)	GTGATCCGGCCTGTTCTTCA	TGACTGGCCGATCCAAAGTC
GAPDH(Homo)	GCATCCTGGGCTACACTG	TGGTCGTTGAGGGCAAT
GAPDH(Mus)	GTCAAGGCTGAGAACGGGAA	AAATGAGCCCCAGCCTTCTC
miR-30a-3p	CGGGCCTTTCAGTCGGATGT	CAGCCACAAAGAGCACAAT
U6	CTCGCTTCGGCAGCACA	AACGCTTCACGAATTTGCGT
FOXK2	GCCACAATCTCTCTCTGAATC	TTCCTGGATGACAGCGGAG

Figure S1

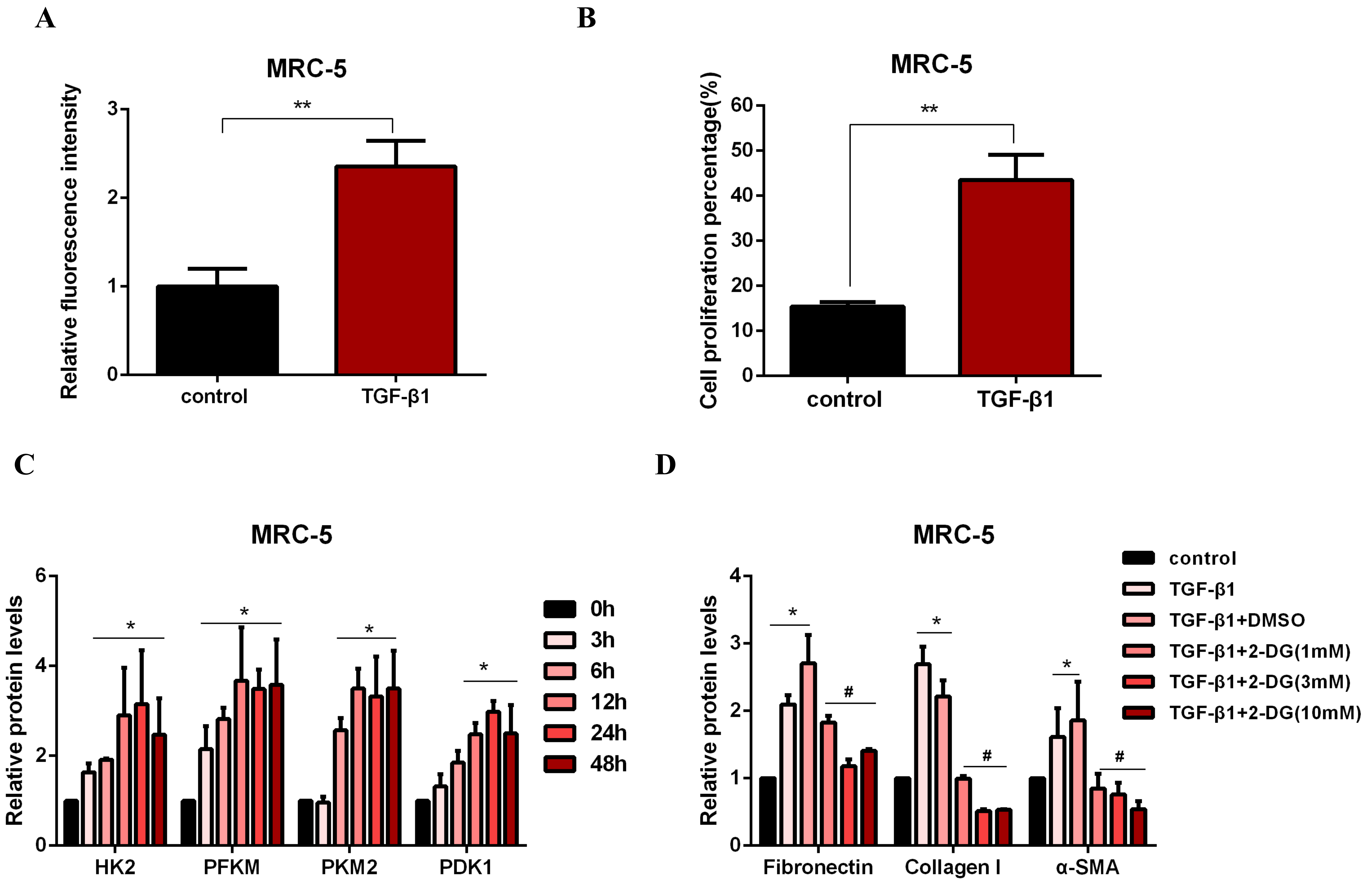

Figure S1. Glycolysis plays a key role in TGF-β1-induced pulmonary fibroblast activation. (A) Relative fluorescence intensity of immunofluorescence staining of α-SMA(*n*=3), ***P*<0.01. (B) Quantification results of EDU staining of MRC-5 cells compared the percentage of cells(*n*=3), ***P*<0.01. (C) Densitometric analysis of HK2, PFKM, PKM2 and PDK1 in MRC-5 cells were treated with 5 ng/ml TGF-β1 for 0h, 3h, 6h, 12h, 24h, 48h(*n*=3), with **P*<0.05 vs. 0h group. (D) Densitometric analysis of Fibronectin, Collagen I and α-SMA in MRC-5 cells for the indicated groups (*n*=3), with **P*<0.05 vs. control group and # *P*<0.05 vs. TGF-β1+DMSO group.

Figure S2

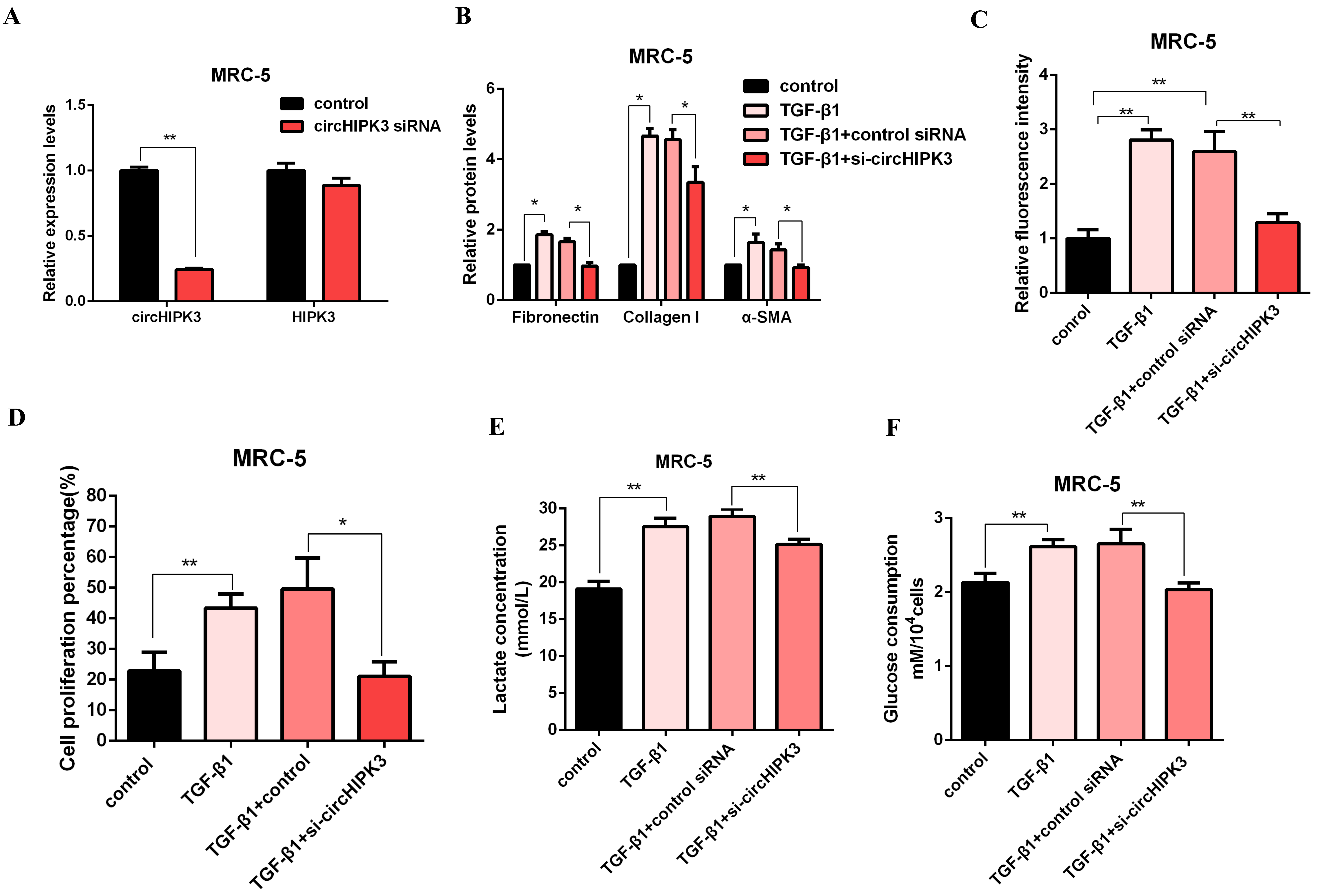

Figure S2. CircHIPK3 is involved in TGF-β1-derived fibroblast activation and proliferation. (A) Expression of circHIPK3 and HIPK3 in nuclear and cytoplasm of MRC-5 were measured via qRT-PCR analysis (*n*=3), ***P*<0.01. (B) Densitometric analysis of Fibronectin, Collagen I and α-SMA in MRC-5 cells for the indicated groups(*n*=3), **P*<0.05. (C) Relative fluorescence intensity of immunofluorescence staining of α-SMA(*n*=3), ***P*<0.01. (D) Quantification results of EDU staining of MRC-5 cells for the indicated groups (*n*=3), **P*<0.05, ***P*<0.01. (E-F) Lactate levels and glucose consumption were determined in MRC-5 cells transfected with control or circHIPK3 siRNA (*n*=3), ***P*<0.01.

Figure S3

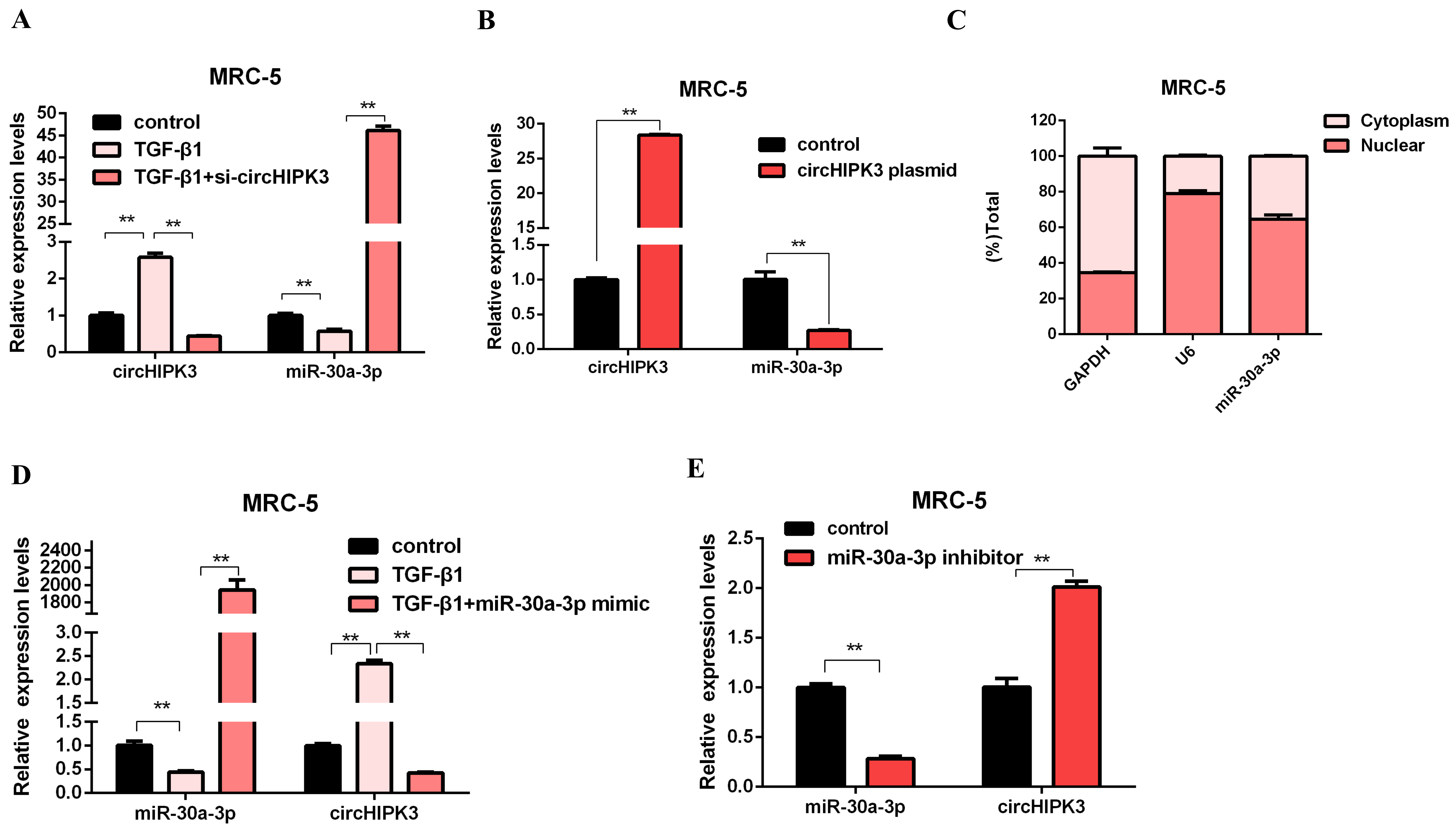

Figure S3. CircHIPK3 acts as a sponge for miR-30a-3p in lung fibroblasts. (A) qRT-PCR were performed to detected the expression of circHIPK3 and miR-30a-3p for control, TGF-β1, and TGF-β1+si-circHIPK3 group (*n*=3), ***P*<0.01. (B) qRT-PCR were performed to detected the expression of circHIPK3 and miR-30a-3p for the control and circHIPK3 plasmid treatment groups (*n*=3), ***P*<0.01. (C) MiR-30a-3p expression was detected in nuclear and cytoplasm RNA extracted from MRC-5 via qRT-PCR. (D-E) qRT-PCR were performed to detected the expression of circHIPK3 and miR-30a-3p for the indicated group (*n*=3), ***P*<0.01.

Figure S4

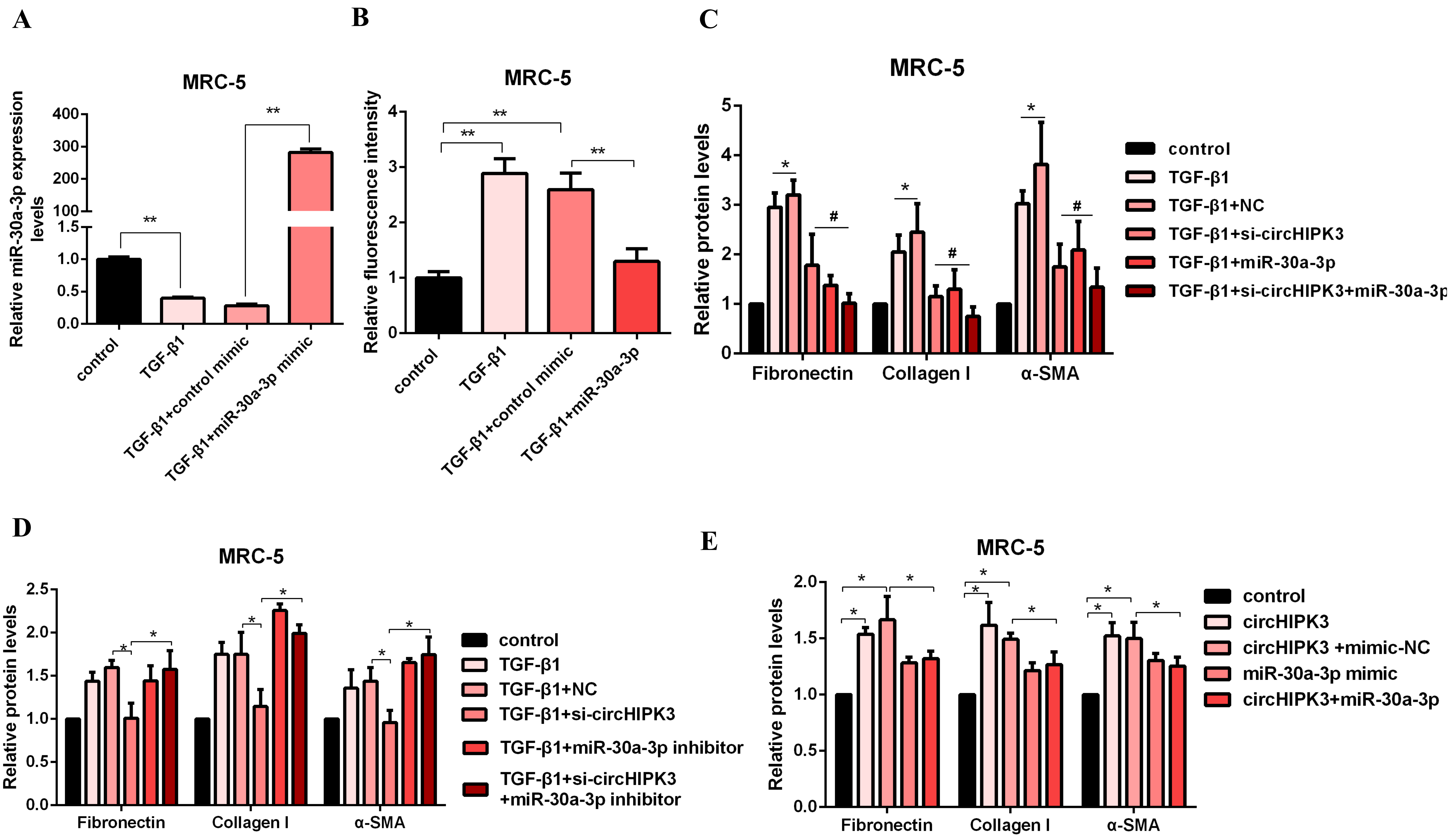

Figure S4. MiR-30a-3p mediates the function of circHIPK3 to regulate fibroblast activation. (A) MiR-30a-3p expression was determined by qRT-PCR in MRC-5 cells for the indicated groups (*n*=3), $**P<0.01$. (B) Relative fluorescence intensity of immunofluorescence staining of Collagen I (*n*=3), $**P<0.01$. (C) Densitometric analysis of Fibronectin, Collagen I and α-SMA in MRC-5 cells for the indicated groups(*n*=3), with $*P<0.05$ vs. control group, #P<0.05 vs. TGF-β1+NC group. (D-E) Densitometric analysis of Fibronectin, Collagen I and α-SMA in MRC-5 cells for the indicated groups(*n*=3), $*P<0.05$.

Figure S5

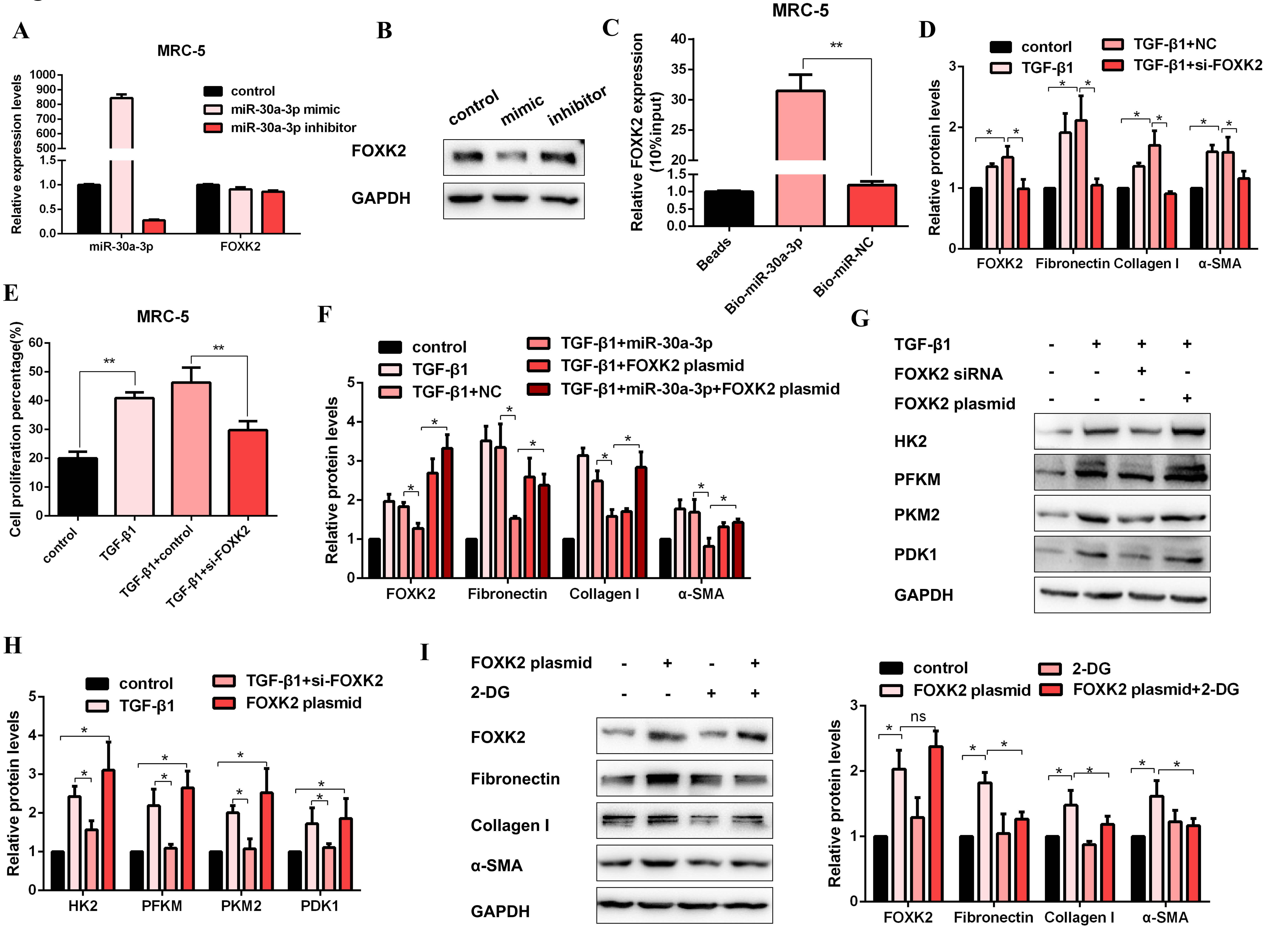

Figure S5. FOXK2 is a functional target of miR-30a-3p and exerts profibrotic effects by regulating glycolysis. (A-B) qRT-PCR and western blot analysis of FOXK2 in MRC-5 cells treated with miR-30a-3p mimic or inhibitor. (C) The relative levels of FOXK2 in MRC-5 cells were pulled down by biotinylated miR-30a-3p. (D) Densitometric analysis of FOXK2, Fibronectin, Collagen I and α-SMA in MRC-5 cells for the indicated groups (n = 3), *P < 0.05. (E) Quantification results of EDU staining of MRC-5 cells for the indicated groups (n=3), **P<0.01. (F) Densitometric analysis of FOXK2, Fibronectin, Collagen I and α-SMA in MRC-5 cells for the indicated groups (n = 3), *P <0.05. (G-H) Western blot and densitometric analysis of HK2, PFKM, PKM2 and PDK1 in MRC-5 cells for the indicated groups (n = 3), *P < 0.05. (I) Western blot and densitometric analysis of FOXK2, Fibronectin, Collagen I and α-SMA in MRC-5 cells for the indicated groups (n = 3), *P < 0.05.

Figure S6

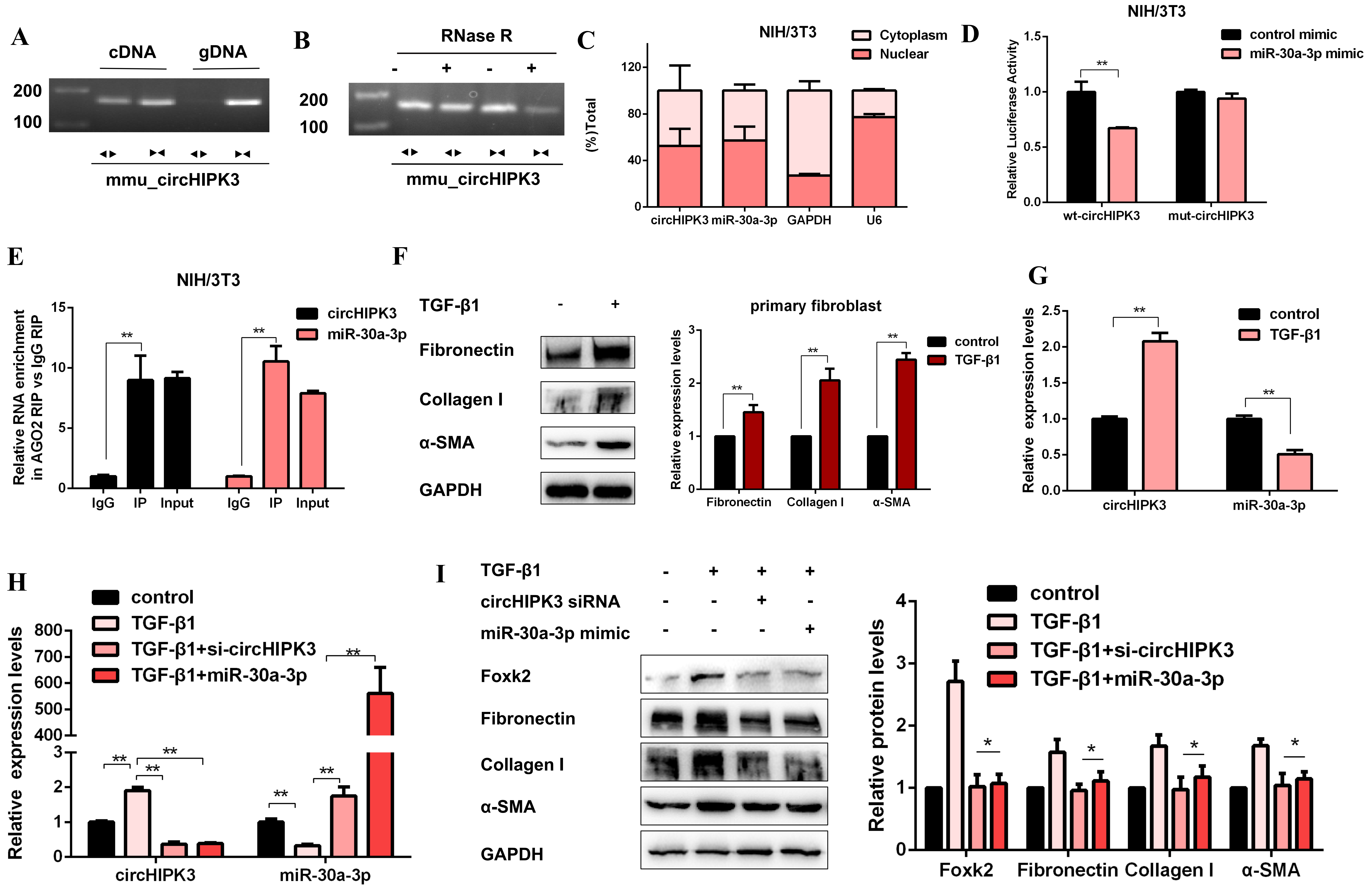

Figure S6. CircHIPK3 acts as a sponge for miR-30a-3p to regulate mouse fibroblast activation. (A) CircHIPK3 was amplified by divergent primers in cDNA but not gDNA of NIH/3T3 via RT-PCR analysis. (B) The expression of circHIPK3 and HIPK3 mRNA in NIH/3T3 was detected by RT-PCR in the presence or absence of RNase R. (C) Expression of circHIPK3 and miR-30a-3p in nuclear and cytoplasm of NIH/3T3 were measured via qRT-PCR analysis. (D) NIH/3T3 were co-transfected with LUC-circHIPK3-wt, or LUC-circHIPK3-mut with miR-30a-3p or scrambled mimic. Luciferase activity was detected 24 h after transfection (n=3), $^{**}P$<0.01. (E) RIP assays for circHIPK3 and miR-30a-3p levels in MRC-5 cells by using AGO2 antibody (n=3), $^{**}P$<0.01. (F)Western blot and densitometric analysis of Fibronectin, Collagen I and α-SMA in mouse primary fibroblasts for the indicated groups (n=3), $^{**}P$<0.01. (G) qRT-PCR analysis of circHIPK3 and miR-30a-3p in mouse primary fibroblasts were treated with 0 or 5 ng/ml TGF-β1 for 48h (n=3), $^{**}P$<0.01. (H) qRT-PCR were performed to detected the expression of circHIPK3 and miR-30a-3p for the indicated group (n=3), $^{**}P$<0.01. (I) Western blot and densitometric analysis of Foxk2, Fibronectin, Collagen I and α-SMA in MRC-5 cells for the indicated groups group (n=3), with$^{*}P$<0.05 vs. the TGF-β1 group.

Figure S7

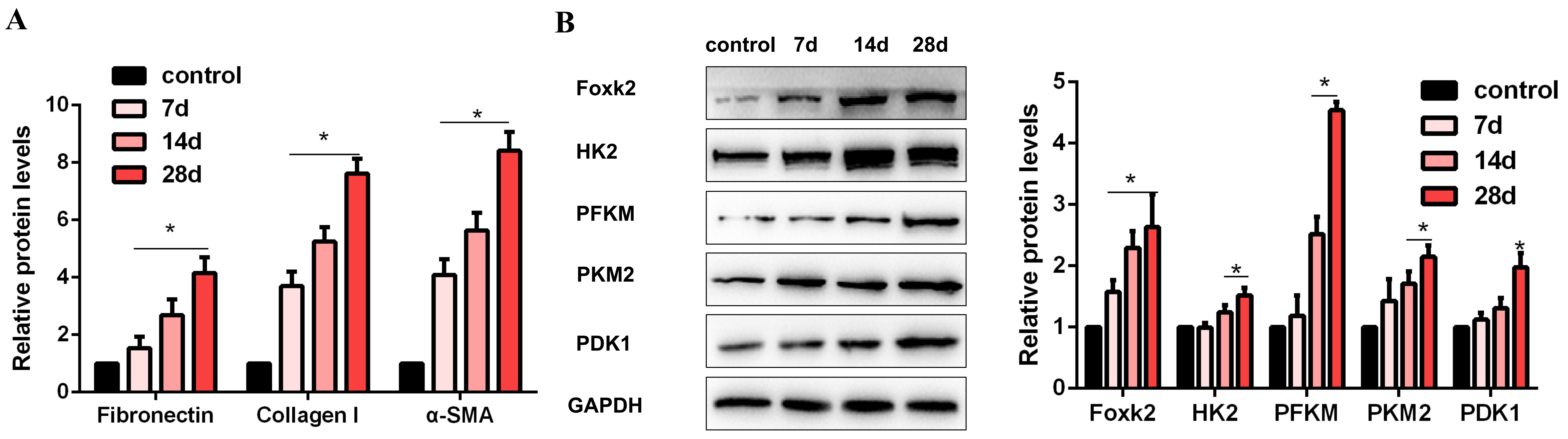

Figure S7. CircHIPK3 and miR-30a-3p are dysregulated during pulmonary fibrogenesis. (A) Densitometric analysis of the protein expression of Fibronectin, Collagen I, and α-SMA in mouse lung tissues (*n*=3), with *P<0.05 vs. the control group. (B) Western blot and densitometric analysis of the protein expression of Foxk2, HK2, PFKM, PKM2 and PDK1 in mouse lung tissues for indicated groups (*n*=3), with *P<0.05 vs. the control group..

Figure S8

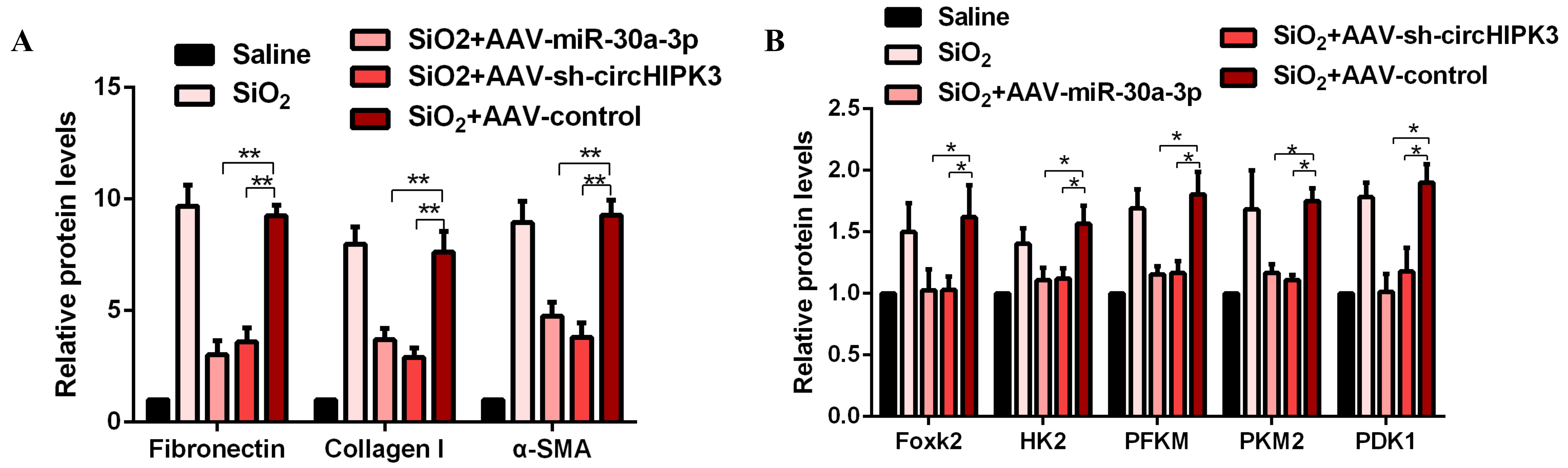

Figure S8. CircHIPK3 and miR-30a-3p regulate silica-induced pulmonary fibrosis in vivo. (A) Densitometric analysis of the protein expression of Fibronectin, Collagen I, and α-SMA in mouse lung tissues groups (*n*=3), **P<0.01. (B) Densitometric analysis of the protein expression of Foxk2, HK2, PFKM, PKM2 and PDK1 in mouse lung tissues for indicated groups (*n*=3), *P<0.05.
